# Effectiveness of Interventions for Prevention of Common Infections Among Opioid Users: A Systematic Review of Systematic Reviews

**DOI:** 10.3389/fpubh.2022.749033

**Published:** 2022-02-22

**Authors:** Svetlana Puzhko, Mark J. Eisenberg, Kristian B. Filion, Sarah B. Windle, Andréa Hébert-Losier, Genevieve Gore, Elena Paraskevopoulos, Marc O. Martel, Irina Kudrina

**Affiliations:** ^1^Department of Family Medicine, Faculty of Medicine and Health Sciences, McGill University, Montreal, QC, Canada; ^2^Jewish General Hospital, Lady Davis Institute for Medical Research, Montreal, QC, Canada; ^3^Department of Epidemiology, Biostatistics and Occupational Health, Faculty of Medicine and Health Sciences, McGill University, Montreal, QC, Canada; ^4^Division of Cardiology, Department of Medicine, Faculty of Medicine and Health Sciences, Jewish General Hospital, McGill University, Montreal, QC, Canada; ^5^Department of Medicine, Faculty of Medicine and Health Sciences, McGill University Health Center, McGill University, Montreal, QC, Canada; ^6^Schulich Library of Physical Sciences, Life Sciences, and Engineering, McGill University, Montreal, QC, Canada; ^7^Queensway Carleton Hospital, Ottawa, ON, Canada; ^8^Department of Anesthesia, Faculty of Medicine and Health Sciences, McGill University, Montreal, QC, Canada; ^9^Faculty of Dentistry, McGill University, Montréal, QC, Canada

**Keywords:** opioid, infection, prevention, intervention, effectiveness, systematic review

## Abstract

**Background:**

The North American opioid crisis is marked by high opioid-related mortality and morbidity, including opioid use-associated infections (OUAIs). Users of pharmaceutical and non-pharmaceutical opioids are at an increased risk of acquiring hepatitis C (HCV), human immunodeficiency virus (HIV), and other infections. No high-level evidence, however, has been synthesized regarding effectiveness of interventions to prevent OUAIs in legal, and illegal/mixed opioid users. The aim of the study is to synthesize available systematic review (SR)–level evidence on the scope and effectiveness of interventions to prevent OUAIs among opioid users.

**Methods:**

A SR of SRs approach was applied. We searched PubMed, Embase, PsycINFO, Cochrane Database of Systematic Reviews, Epistemonikos and Google Scholar from inception to September 2020. Data selection and extraction were performed independently by three researchers. Risk of bias and quality of evidence were assessed using the AMSTAR2 tool. Results were narratively synthesized. Strength of evidence for each category was reported.

**Results:**

Eleven of twelve identified SRs included interventions to prevent HCV/HIV transmission in persons who inject drugs (PWID), including opioids. One SR evaluated interventions to prevent recurrent infectious endocarditis. There was sufficient and tentative SR of SRs-level evidence for the effectiveness of opioid substitution therapy (OST) in preventing HIV and HCV, respectively. We found tentative evidence to support effectiveness of needle/syringe exchange programs (NSP) in HIV prevention, and sufficient evidence to support effectiveness of the combined OST and NSP in HCV prevention. There was insufficient SR-level evidence to support or discount effectiveness of other interventions to prevent OUAIs. No SR focused on non-PWID populations.

**Conclusion:**

SR-level evidence supports the use of OST, NSP, and combined interventions for the reduction of HCV and HIV transmission in PWID. More research on prevention of other OUAIs and on prevention of OUAIs in non-PWID populations is urgently needed.

**Systematic Review Registration:**

Registered in PROSPERO on July 30, 2020. https://www.crd.york.ac.uk/prospero/display_record.php?RecordID=195929, identifier: #195929.

## Introduction

The North American opioid crisis is a major public health emergency (1–3). Half a million lives have been lost to the opioid crisis over the past two decades in the US (4). Since January 2016, more than fifteen thousand apparent opioid-related deaths were reported in Canada (5).

Although opioid-induced morbidity has not gained as much attention as opioid-associated mortality, it also constitutes serious health and economic burden. In addition to being acquired via sharing contaminated equipment by illegal users and by risky behavior like unprotected sex, it is theorized that OUAIs entry could be facilitating secondary immunosuppressive properties of many opioids. Users of illegally obtained pharmaceutical (approved for medical purposes in humans) (6) or non-pharmaceutical opioids, especially persons who inject drugs (PWID), are at an increased risk for serious bacterial infections (infective endocarditis, osteomyelitis, septic arthritis, pneumonia, meningitis, cellulitis, abscesses etc.) requiring lengthy treatment courses and expensive hospital stays (7–9). Commonly discussed examples of viral OUAIs in PWID are Hepatitis C (HCV) and human immunodeficiency viruses (HIV). Both infections are associated with a substantial burden for individuals and society. Importantly, patients who use pharmaceutical opioids as prescribed by physician appear to be more susceptible to viral, bacterial, and fungal infections when compared to patients not treated with opioids (10–15). Continuous use of high-dose opioids may accelerate viral (hepatitis A, B, C, and HIV) entry and replication and increases the risk of infections (11, 13).

Many OUAIs, including HCV and HIV, are preventable (16). In the population of persons who inject drugs, including opioids, a guideline from the Center for Disease Control and Prevention (16) recommends implementing preventive measures and screening for common viral infections such as HCV, HIV, hepatitis B virus, Herpes Simplex Virus type 2, Human Papillomavirus, tuberculosis and common sexually transmitted diseases. Likewise, the Best Practice Recommendations for Canadian Harm Reduction Programs (17) emphasize the importance of routine skin care to prevent bacterial and fungal infections in PWID. It remains, however, unclear what interventions to prevent OUAIs should be recommended in non-drug injecting populations and in legal or mixed (see Methods, Definitions section) users of opioids. It is, therefore, imperative to evaluate the scope of existing interventions and their relative effectiveness, to identify knowledge gaps, and propose recommendations for future strategies.

To identify potential scope and types of interventions aimed to prevent problems associated with the opioid crisis, an initial search for an overarching research project (Canadian Institutes of Health Research [CIHR] grant #EOC-162067) was performed in 2019. Among others, this search produced 18 systematic reviews (SRs) (Appendix 1) related to OUAIs prevention. All identified studies, however, appeared to be limited to HCV and HIV prevention in the population of PWID. In addition, in several SRs, the use or co-use of opioids was not explicitly confirmed.

To account for the above, this systematic review of SRs (SR of SRs) was designed to capture a broader scope of preventive interventions with evaluated effectiveness by including all types of opioids users (legal, illegal, and mixed), pharmaceutical and non-pharmaceutical opioids, and all routes of use. Our review question was “What is the SR-level evidence on the scope and effectiveness of interventions to prevent common infections in people who use opioids?”

The specific objectives were: (1) to describe SR-level evidence for the scope of interventions with evaluated effectiveness in prevention of OUAIs; (2) to synthesize the SR-level evidence on the effectiveness of interventions in preventing OUAIs; and (3) to identify knowledge gaps in this area. With a growing literature on a variety of infections and immunosuppression associated with the use of opioids, of special interest for the authors was to identify the evidence on the effectiveness of interventions to prevent OUAI in the population other than PWID and on infections other than HCV and HIV. In light of the worsening opioid crisis and overstretched healthcare resources, synthesizing the highest level (SR-level) of evidence is a timely and necessary effort to inform knowledge users and policy/decision makers about the effectiveness of OUAIs preventive strategies and existing knowledge gaps in this area.

## Methods

To address the review question, we synthesized SR of SRs-level evidence on the scope and effectiveness of interventions to prevent OUAIs. The detailed description of Methods and our PICO question, including all elements, are provided in the Appendix 5. One can also refer to the published study protocol (18). Our PICO question is: What is the SR-level evidence on the scope and effectiveness of interventions to prevent opioid use associated infections in adults who use legal/illegal opioids as compared to those not participating in an intervention or as compared to the time prior to the intervention implementation?

### Definitions

For the purposes of this SR of SRs, we roughly categorized opioid use into legal and illegal/mixed opioid consumption. Opioid use was defined as any opioid use via any route of administration. This includes opioid co-use with other substances. Legal use was defined as the use of therapeutically prescribed pharmaceutical opioids (6) by the person to whom it was prescribed. Pharmaceutical opioids are opioids produced by a pharmaceutical company and approved for medical purposes in humans (5). Opioid use disorder (19), opioid misuse, and mixed use of pharmaceutical and/or non-pharmaceutical opioids were classified as illegal/mixed opioid use. Persons who use both pharmaceutical and non-pharmaceutical opioids or those who use opioids both legally and illegally were defined as mixed users.

### Study Design and Search Strategy

We applied an SR of SRs approach (20), a subtype of the Overview of Systematic Reviews (21) methodology. The difference between the SR of SRs methodology and other types of the Overview of Reviews is described in the Appendix 5. The choice of SR of SRs methodology was selected based on the results of the initial search for the overarching project, which identified multiple SRs related to our review question. A protocol for this SR of SRs was developed following the Cochrane Handbook for Systematic Reviews of Interventions, Chapter 5 (21), Cochrane Handbook for Systematic Reviews of Interventions Version 510 (22) and using the Joanna Briggs Institute Reviewer's Manual (23). The results are reported according to the Preferred Reporting Items for Systematic Reviews and Meta-analyses (PRISMA) and Preferred Reporting Items for Overview of Systematic Reviews (PRIO-harms) statements (Please see PRISMA 2020 checklist, Appendix 2).

The protocol was registered in PROSPERO on July 30, 2020 (#195929).

We conducted a systematic search of PubMed, Ovid Embase, Ovid PsycINFO, Cochrane Database of Systematic Reviews, Epistemonikos and Google Scholar databases from inception to February 2020, with the search updated in September 2020. The search strategy was based on the following 4 concepts joined by the Boolean operator “AND”: (1) opioids (all commonly used opioids, including generic and brand names, and synonyms); (2) infections (all common viral and bacterial infections associated with opioid use); (3) preventive interventions (all interventions designed to prevent infections in opioid users) and (4) systematic reviews with or without meta-analysis. The complete search strategy is presented in Appendix 4. A medical liaison librarian (GG) was engaged in designing the search strategy.

Included studies were SRs synthesizing studies of interventions to reduce or prevent infection transmission/acquisition among users of pharmaceutical or non-pharmaceutical opioids by any route of use, and reporting on the effectiveness of the interventions, with or without meta-analysis. A publication was considered an SR if it was reported according to the Preferred Reporting Items for Systematic Reviews and Meta-analysis (PRISMA) checklist and met the following criteria: (1) described methods, comprising a systematic search strategy and criteria for inclusion/exclusion; (2) used a comprehensive search in all relevant databases and an exhaustive search strategy; (3) performed a formal quality assessment of included studies applying a validated tool (e.g., Jadad, Cochrane RoB). Only reviews with retrievable full text articles in English or French were included. SRs that synthesized information on the studies not relevant to the North American context (e.g., humanitarian programs) were excluded. Conference abstracts were not eligible for inclusion. Only studies where outcome of the intervention/s were clearly identified and the effectiveness of interventions was evaluated were included. SR that synthesized information of all study types (e.g., experimental or observational) either with a comparator group (opioid users not participating in an intervention/program/not affected by a policy/approach) or time/population prior to implementation of policy (for population-level studies) were included.

### Outcomes

The outcome of Objective 1 was the scope of interventions for prevention of infections in opioid users. The outcome of Objective 2 was effectiveness of interventions to prevent infections in opioid users, potentially appropriate for the North American context. Measures of effectiveness of the intervention/program/policy were effect measures of an association between infection/disease incidence (e.g., HCV/HIV seroconversion) and participation in the intervention, estimated by either odds ratio, risk ratio, or hazard ratio in participants or groups. For SRs with meta-analysis, pooled effect measures were reviewed. For SRs without meta-analysis, effect measures reported by original studies were extracted. The outcome of Objective 3 was the identification of knowledge gaps in the studied area.

### Study Selection and Data Extraction

First, titles and abstracts of all records identified through the systematic search were screened by three members of the research team (SP, IK, EP) independently. Any potentially relevant study identified by either reviewer was carried forward. Subsequently, full texts of potentially eligible publications were reviewed independently by SP and IK, with disagreements resolved by consensus. The publications remaining after this full text review were included. The RAYYAN platform (available at McGill: https://libraryguides.mcgill.ca/rayyan#s-lg-box-13326907) and an Excel spreadsheet were used to record decisions and reasons for exclusion. The “Endnote” bibliographic software (Endnote X8.1) was used for duplicates removal and storage.

Two researchers (SP and IK) independently extracted the data using a standardized, pilot-tested data collection form, according to Cochrane recommendations for overviews of SRs (21). In case of unclear or missing data, authors were contacted. In overlapping reviews, if any SR contained important information not included in other reviews, the most complete review was chosen for inclusion. Disagreements were resolved by consensus, with assistance of the third reviewer (EP) when necessary.

### Risk of Bias (Quality) Assessment

Risk of bias/methodological quality of included SRs was assessed using the AMSTAR 2 tool (24). The critical domains appraised (24) are shown in Table 1. Overall quality of the results of SRs was graded as either high, moderate quality, low, or critically low. The quality assessment was performed by two independent reviewers (SP and IK). Disagreements were resolved by discussion and consensus, with the help of a third reviewer (EP) when necessary. SRs were categorized into high quality “core” reviews, which produced the essential evidence on the effectiveness of interventions, and “supplementary” reviews. This method of categorization (Appendix 5) was previously described and used in the published overviews of SRs related to our topic (37, 38). The level of evidence was categorized as “sufficient”, “tentative”, “insufficient” or “no” SR-level evidence, as described in Appendix 5, using a previously developed framework (38, 39).

**Table 1:** Evaluation quality of evidence of included SRs using AMSTAR2.

**N**	**Systematic review: References**	**1**	**2**	**3**	**4**	**5**	**6**	**7**	**8**	**9**	**10**	11^*^	12^*^	**13**	**14**	15^*^	**16**	**Quality**
1	Bahji et al. (25)	Y	Y	Y	Y	Y	Y	N	Y	Y	N	NC	NC	Y	Y	NC	Y	High
2	Davis et al. (26)	Y	Y	Y	PY	Y1	Y	N	Y	PY	Y	Y	Y	Y	Y	Y	Y	High
3	Platt et al. (27)	Y	Y	Y	Y	Y	Y	N	Y	Y	Y	Y	Y	Y	Y	Y	Y	High
4	Sawangjit et al. (28)	Y	Y	Y	PY	N	Y	N	Y	Y	N	Y	Y	Y	Y	Y	Y	Moderate
5	Aspinal et al. (29)	Y	N	N	PY	Y	N	N	PY	NA	Y	N	Y	Y	Y	Y	Y	Moderate
6	Underhill et al. (30)	Y	Y	Y	Y	Y	Y	N	N	Y	N	NC	NC	N	N	NC	Y	Moderate
7	Abdul-Quader et al. (31)	Y	N	Y	PY	Y	Y	N	PY	N	N	NC	NC	Y	Y	NC	Y	Low
8	MacArthur et al. (32)	Y	N	Y	Y	Y1	Y	N	Y	Y	N	Y	Y	Y	Y	Y	Y	Moderate
9	Sacks-Davis, (33)	Y	N	N	Y	Y	N	N	Y	Y	N	NC	NC	Y	Y	NC	Y	Moderate
10	Gowing et al. (34)	Y	Y	Y	Y	Y2	Y	Y	Y	Y	Y	NC	NC	Y	Y	NC	Y	High
11	Hagan et al. (35)	Y	Y	Y	Y	Y2	Y	N	PY	N	N	Y	N	N	Y	Y	Y	Low
12	Jones et al. (36)	Y	N	Y	Y	Y1	N	N	Y	N	N	NC	NC	N	N	NC	Y	Critically low

*Y, Yes; N, No; PY, Partial Yes; NC, Not Conducted*.

* *Domains assessing the methodological quality of meta-analyses*.

*Critical domains pertaining the overall quality of included systematic reviews*.

*Item 1: Did the research questions and inclusion criteria for the review include the components of PICO?*

*Item 2: Did the report of the review contain an explicit statement that the review methods were established prior to the conduct of the review and did the report justify any significant deviations from the protocol?*

*Item 3: Did the review authors explain their selection of the study designs for inclusion in the review?*

*Item 4: Did the review authors use a comprehensive literature search strategy and a justification provided when there are language restrictions?*

*Item 5: Did the review authors perform study selection in duplicate?*

*Item 6: Did the review authors perform data extraction in duplicate?*

*Item 7: Did the review authors provide a list of excluded studies and justify the exclusions?*

*Item 8: Did the review authors describe the included studies in adequate detail?*

*Item 9: Did the review authors use a satisfactory technique for assessing the risk of bias (RoB) in individual studies that were included in the review?*

*Item 10: Did the review authors report on the sources of funding for the studies included in the review?*

*Item 11: If meta-analysis was justified did the review authors use appropriate methods for statistical combination of results?*

*Item 12: If meta-analysis was performed did the review authors assess the potential impact of RoB in individual studies on the results of the meta-analysis or other evidence synthesis?*

*Item 13: Did the review authors account for RoB in individual studies when interpreting/ discussing the results of the review?*

*Item 14: Did the review authors provide a satisfactory explanation for, and discussion of, any heterogeneity observed in the results of the review?*

*Item 15: If they performed quantitative synthesis did the review authors carry out an adequate investigation of publication bias (small study bias) and discuss its likely impact on the results of the review?*

*Item 16: Did the review authors report any potential sources of conflict of interest, including any funding they received for conducting the review?*

*The colors reflect the quality of evidence of included SRs*.

*Green color indicates high quality of evidence*.

*Orange color indicates moderate quality of evidence*.

*Light red color indicates low quality of evidence*.

*Red color indicates critically low quality of evidence*.

### Data Synthesis

Overlap of the primary studies was mapped by providing a citation matrix (20) (Table 2). The number of overlapping studies and their contribution to the analysis (20, 21) were narratively described. The extracted data were tabulated according to the types of interventions and the types of infections (Tables 3, 4). We performed qualitative analysis of findings by narrative synthesis. If opioid use was confirmed for only one primary study of all studies included in the SR, the results were extracted and discussed but not included in the data synthesis. In addition, for the interventions for which SR-level evidence was sparse, the results of primary studies were discussed but were not considered as an SR-level evidence. The results were summarized in Tables 5, 6 and in **Figure 3** and presented as a Supplementary Figure 1. Knowledge gaps and further research directions were identified.

**Table 2:** Citation matrix.

**SR**	**Overlapping references**																				**Non-overlapping references**												
Abdul-Quader et al. (31)																		B11a	B11b	DJ05a	S99	DJ07	T11	DJ09	DJ09	G01	RJ06	HDJ06	H05	DJ10	DJ05b	G98	
Hagan et al. (35)	H95*	VDB07	H04	H09	P01	R07	G07	AS08**	S09	T02	C09	B/L04	R96	T00	VB98	C97	K10				B00	D05	L97	Hal04	M06	H03	H10	H02	K02	S03			
MacArthur et al. (32)	M93	VDB07									W92										B12	J12-	V01	S09	C95	N02	K06						
Sawangjit et al. (28)																					T03	M02	G95	B10	N08	V09	B06						
Gowing et al. (34)	M93										W92										M94	S94											
Platt et al. (27)	H95	VDB07		H09	P01	R07				T02	C09	B/L04	R96	T00	VB98	C97					A/P14	J15	M15	W14	T14	H11	H15	S12	V15	N14	P15	H99	M15-
Davis et al. (26)	H95		H04	H09	P01	R07																T15											
Sacks-Davis, (33)							G07	AS08	S09																								
Jones et al. (36)		VDB07																															
Aspinal et al. (29)		VDB07															K10	B11a	B11b	DJ05a	V01	M00	S99	B97	S96	DJ96a	DJ96b						
Underhill et al. (30)																					D09												
Bahji et al. (25)																					H10	P12	R16										

*Green color: “O+” (participants are opioids-users); orange color: “O”-(participants are drug users but the use of opioids is not specified); red color: study included due to non-relevant country; blue color: full text non-available*.

*Each abbreviation includes the first name of the author and the last two digits of the year the paper was published, e.g. “H95” is for “Hagan et al., 1995”. The full list of included papers is provided in the Appendix 1*.

*This study was not included in the behavioral intervention analyses*.

**Table 3:** Characteristics of included systematic reviews.

**Characteristics**	**Number of SRs (*N* = 12)**
Type of SRs	
Core	8
Supplemental	4
Number of primary studies	84
Relevant to North American context	82
Full text available	82
Overlap of primary studies	
Overlapping between SRs	20
Not overlapping between SRs	64
Opioid use in study participants	
Confirmed use/co-use	68
Unconfirmed use/co-use	12
Types of evaluated interventions	
Opioid substitution therapy	4
Needle and syringe exchange program	6
Opioid substitution therapy+ Needle and syringe exchange program	2
Syringe disinfection	1
Behavioral interventions	3
Multi-component interventions	1
Outpatient antibiotics treatment	1
Addiction services	1
Participants	
PWID	12
Non-PWID	0
Settings	
Participants of harm reductions programs/location of social services programs	10
Primary care settings	1
Prison	
Infections to prevent	
HCV	4
HIV	2
HCV and HIV	3
Infectious endocarditis	1
Other opioids use associated infections	0

*SR, systematic reviews; PWID, persons who inject drugs; HCV, hepatitis C virus; HIV, human immunodeficiency virus*.

**Table 4:** Summary of core and supplementary reviews of the effectiveness of interventions for preventions of common infections in opioid users.

**#**	**References**	**Title**	**Dates covered**	**P**	**I**	**C**		**O**	**Type of review**	**Number of relevant studies included in the review, confirmed/ unconfirmed use of opioids**
				**Population**	**Interventions covered in review**	**Infection**	**Comparator**	**Outcome measure**		
1	Bahji et al. (25)	Harm Reduction for Injection Drug Users with Infective Endocarditis: A Systematic Review	Up to 2020	Adult patients, primarily males, mean age 39 y.,with injection drug use-related infectious endocarditis in the context of opioid use disorder;	1) Skin and needle hygiene educational intervention 2) Addiction services 3) 18 days of outpatients parenteral antibiotic treatment (OPAT)	Infectious endocarditis	1) no intervention group 2) self-control 3) self-control	1) HR for recurrent endocarditis 2) % recurrent endocarditis 3) % readmitted for infective complications	Core	1) 1 O+; 2) 1 O+; 3) 1 O+
										
2	Davis et al. (26)	Needle exchange programs for the prevention of hepatitis C virus infection in people who inject drugs: A systematic review with meta-analysis	Up to 2016	Adult young (<40 y.) PWID enrolled in harm reduction programs, emergency rooms, county health department, jails, streets, social service agencies	NEP	HCV	NEP non-users	HR or OR, for HCV seroconversion, lab confirmed	Core	2 O+; 3 O?
										
3	Platt et al. (27)	Needle syringe programmes and opioid substitution therapy for preventing hepatitis C transmission in people who inject drugs	Up to 2015	Adult PWID, recruited by street outreach, respondent-driven sampling/ service attenders/ combination of both	Opioid Substitution Therapy (OST)+Needle Syringe Programmes (NSP)	HCV	1) OST vs. no OST 2) ([High NSP coverage] + [no OST]) vs. (low coverage NSP) 3) (Low NSP coverage + [no OST]) vs.no NSP 4) ([High/low NSP coverage] + OST) vs. ([no OST] + [low/no coverage NSP])	OR, RR, IRR, HR for HCV seroconversion, lab confirmed	Core	NSP: 3 O+, 3 O?; OST: 13 O+; NSP+OST : 6 O+
4	Sawangjit et al. (28)	Effectiveness of pharmacy-based needle/syringe exchange programme for people who inject drugs: a systematic review and meta-analysis.	Up to 2016	Adult PWID, mean age 30–40 y., recruited in primary care settings	NSP	HCV and HIV	1) Pharmacy-based NSP vs. non-pharmacy-based NSP 2) Pharmacy-based NSP vs. no NSP 3) Van-based NSP vs. no NSP	OR for decline in HCV/HIV prevalence	Core	1) 3 O+, 1 O?; 2) 2 O+, 1 O?; 3) 1 O+
										
5.	Aspinall et al. (29)	Are needle and syringe programmes associated with a reduction in HIV transmission among people who inject drugs: a systematic review and meta-analysis	Up to 2012	PWID; for 5 studies characteristics are not described; for the remaining studies, adults, mean age 29–37 y.), HIV negative at baseline in cohort studies	≤100% syringes from any safe source	HIV	PWID who were not, or were less frequently, exposed to NSP	HIV incidence (OR, HR, RR) by serological testing	Core	11 O+; 1 O?
										
6	Underhill et sl. (30)	HIV prevention for adults with criminal justice involvement: A systematic review of HIV risk-reduction interventions in incarceration and community settings	Up to 2014	Adults, age≥ 18 y., with criminal justice involvement	Methadone maintenance treatment (MMT), prison-based, duration <18 days - >237 days	HIV	No treatment	HR for HIV seroconversion	Supplemental, specific population	1 O+
7	Abdul-Quader et al. (31)	Effectiveness of structural-level needle/syringe programs to reduce HCV and HIV infection among people who inject drugs: a systematic review	Up to 2011	Adult PWID, age≥18 y., PWID, recruited at street gathering for PWID, harm reduction and drug treatment programs	NSP on a public health scale, with distribution of ≥ 10 needles/syringes per PWID per year and ≥ 50 % coverage of PWID population	HCV/HIV	Individuals or groups received the intervention vs. those who did not, or a comparison of individuals or groups before and after receiving the intervention.	HCV/HIV incidence or prevalence, lab. confirmed	Supplemental	11 O+; 4 O?
										
8	MacArthur et al. (32)	Opiate substitution treatment and HIV transmission in people who inject drugs: systematic review and meta-analysis	Up to 2011	Adult PWID, median age 26–39 y., recruited at drug treatment clinics, community settings and outreach programs	1) Methadone maintenance treatment (MMT); 2) Methadone detoxication treatment (MD)	HIV	1) non-participants 2) MD vs. MMT	RR for HIV seroconversion, lab confirmed	Core	10 O+
										
9	Sacks-Davis et al. (33)	Behavioral interventions for preventing hepatitis C infection in people who inject drugs: a global systematic review	Up to 2010	Adult PWID, mean age 24–41 y., from ever injected to injected ≥1/past 6 months	Behavioral interventions: 1) Peer-educator training 2) Counseling	HCV	Non-participants	HCV incidence: either RR or cumulative incidence	Core	1) 2 O+; 2) 1 O+
10	Gowing et al. (34)	Oral substitution treatment of injecting opioid users for prevention of HIV infection	Up to 2011	Adult PWID (age differed between the studies), or people with a recent history (last 3 months) of injecting drug use at the time of entry	OST	HIV	1) before vs. after treatment 2) participants vs. non-participants 3) vs. IDU receiving treatment not involving administration of an opioid agonist.	Changes in HIV incidence rate in relation to intervention	Core	4 OST O+
										
11	Hagan et al. (35)	A systematic review and meta-analysis of interventions to prevent hepatitis C virus infection in people who inject drugs.	Up to 2010	Adult PWID and non–injection drug users, participants of harm-reduction programs	1) Drug-treatment programs (non-specified and OST); 2) Syringe-access programs; 3) Syringe disinfection with bleach; 4) Individual behavioral interventions; 5) Combinations of any of these services	HCV	Most studies: participation vs. non-participation.	OR, RR, HR of HCV seroconversion, lab confirmed	Supplemental	1) 12 O+, 1 O?; 2) 4 O+, 3 O?; 3) 1 O+, 3 O?; 4) 1 O+; 5) 1 O+
										
12	Jones et al. (36)	Optimal provision of needle and syringe programmes for injecting drug users: A systematic review	Up to 2008	Adult (mean age 29–30 y.) ever PWID, volunteer participants of Amsterdam Cohort Study of PWID	OST +NSP (full or incomplete participation)	HCV, HIV	Participants vs. non-participants	HCV/HIV incidence or prevalence	Supplemental	1 O+

*PWID, persons who inject drugs; OPAT, outpatients antibiotic treatment; NSP, needle and syringe programmes; NEP, needle exchange programmes; OST, opioid substitution treatment programme; MMT, methadone maintenance treatment; MD, methadone detoxication treatment; LAAM, levacetylmethadol; “O+” (confirmed use of opioids) or “O?” (unconfirmed use of opioids) in the primary studies*.

**Table 5:** Summary of results for needle/syringe exchange and opioid substitution treatment to prevent HCV and HIV infections in opioid users.

**References**	**Infection**	**Method of analysis**	**Results**	**Conclusion of SR**	**Quality of SR by AMSTAR2**	**Evidence statement (Conclusion of SR of SRs)**
**NSP**						
Davis et al. (26)	HCV	Meta-analysis, random effects model	Pooled OR = 0.51, 95% CI: 0.05, 5.15 (2 studies); pooled HR: 2.05, 95% CI: 1.39, 3.03 (4 studies)	The impact of NSP on the prevention of HCV in PWID remains unclear, likely due to substantial between-study heterogeneity	High	Tentative evidence to discount NSP effectiveness in preventing HCV as a sole intervention
Platt et al. (27)	HCV	Meta-analysis, random effects model	High NSP coverage vs. (no/ low NSP coverage): pooled RR = 0.79, 95% CI: 0.39, 1.61) (5 studies)	It is unclear whether high coverage NSP (defined as regular attendance, or as “all injections being covered by a new needle/syringe”) reduces the risk of HCV infection across all studies globally	High	
Hagan et al. (35)	HCV	Meta-analysis, random effects model	Pooled RR = 1.62; 95% CI: 1.04, 2.52 (7 studies)	Most studies reported no significant risk reduction	Low	
Aspinell et al. (29)	HIV	Meta-analysis, random effects model	HIV transmission RR = 0.66, 95% CI: 0.43, 1.01 for 12 studies, and RR = 0.42, 95% CI: 0.22, 0.81 for 6 higher quality studies	NSP is effective in reducing HIV transmission in PWID, likely in combination with other harm reduction interventions. NSP should be considered as just one component of a programme of interventions to reduce HIV risk.	Moderate	Tentative evidence to support NSP effectiveness in preventing HIV as a sole intervention or a component in the multicomponent intervention
Sawangjit et al. (28)	HCV/HIV	Meta-analysis, random effects model	HCV prevalence: Pharmacy-based NSP vs no NSP OR = 0.26, 95% CI: 0.18, 0.38 (2 studies), after removal of a seriously biased studies NA HIV prevalence: Pharmacy-based NSP vs no NSP OR = 0.56, 95%CI: 0.18, 1.7 (3 studies), after removing seriously biased studies OR = 0.80, 95% CI: 0.52, 1.22 (1 study)	The effect of pharmacy-based NSP on HIV/HCV prevalence is unclear.	Moderate	
Abdul-Quader et al. (31)	HCV/HIV	Narrative synthesis	15 studies reported effectiveness of structural-level NSP to reduce HIV or HCV prevalence/ incidence; 9 studies reported decreases in HIV prevalence, 6 reported decrease in HCV prevalence, and 3 reported decreases in HIV incidence.	The results support effectiveness of NSP as a structural-level intervention to reduce population-level HCV/HIV infection	Low	
**OST**						
Hagan et al. (35)	HCV	Meta-analysis, random effects model	Substance use treatment, non-specified: RR = 1.21, 95% CI: 0.71, 2.08 (5 studies); Substance use treatment, ORT: RR = 0.60, 95% CI: 0.35, 1.03 (8 studies)	The impact of ORT on HCV seroconversion is inconsistent	Low	Tentative evidence to support OST/ORT effectiveness in preventing HCV
Platt et al. (27)	HCV	Meta-analysis, random effects model	Current OST vs. no OST: pooled R = 0.50, 95%CI: 0.40, 0.63 (12 studies)	Current use of OST (defined as use at the time of survey or within the previous six months) may reduce risk of HCV acquisition by 50%.	High	
MacArthur et al. (32)	HIV	Meta-analysis, random effects model	MMT: RR = 0.46, 95%CI: 0.32, 0.67 (9 studies) MD: No evidence for association with a reduction in the risk of HIV transmission.	OST is important in HIV prevention in persons who inject (opiate) drugs.	Moderate	Sufficient evidence to support OST effectiveness in preventing HIV
Gowing et al. (34)	HIV	Narrative synthesis	All 4 studies showed lower rates of HIV seroconversion associated with OST	OST is associated with consistently lower rates of HIV seroconversion	High	
**Combined OST and NSP**						
Platt et al. (27)	HCV	Meta-analysis, random effects model	For (OST + [high/low NSP]) vs. ([no OST] + [low/no NSP]) RR = 0.45, 95%CI: 0.22, 0.94 (3 studies) For (OST + [high NSP]) vs. ([no OST] + [low/no NSP]) RR = 0.26, 95%CI: 0.07, 0.79 (3 studies)	The combined use of high coverage NSP with OST may reduce risk of hepatitis C infection by 74%	High	Sufficient evidence to support effectiveness of OST+NSP in preventing HCV Insufficient evidence to support OST+NSP in preventing HIV
Jones et al. (36)	HCV/HIV	NA (only 1 relevant study)	Full participation, compared to no harm reduction: For HIV, IRR = 0.32; 95% CI: 0.17, 0.62, for HCV IRR = 0.15; 95% CI: 0.06, 0.40. Incomplete participation, compared to no harm reduction: For HIV, IRR = 0.74; 95% CI: 0.43, 1.27; for HCV: IRR = 1.04; 95%CI: 0.53, 2.05 (1 study)	Based on 1 primary study, the combination of methadone treatment and full participation in NSP is effective in reducing HIV/HCV incidence in drug users	Critically low	
**Multi-component interventions**						
Hagan et al. (35)	HCV	Meta-analysis, random effects model	Multicomponent interventions, 2 studies with heterogenous types of MCI: 1 study OSP+Behavioral intervention, 1 study OST+NSP RR = 0.25, 95%CI: 0.07, 0.83	Combined interventions were effective at reducing HCV seroconversion.	Low	Insufficient SR-level evidence to support effectiveness of combined OST and behavioral intervention in preventing HCV (only 1 primary study)

**Table 6:** Summary of results of systematic reviews evaluating effectiveness of other interventions to prevent infections in opioid users.

**References**	**Infection**	**Method of analysis**	**Results**	**Conclusion of SR**	**Quality of SR by AMSTAR2**	**Evidence statement (Conclusion of SR of SRs)**
**Syringe disinfection (SD)**						
Hagan et al. (35)	HCV	meta-analysis, random effects model	Pooled RR=1.08, 95% CI: 0.66, 1.75 (4 studies)	No effect on HCV transmission, consistent between all studies	Low	Insufficient evidence to support or discount effectiveness of syringe disinfection with bleach in preventing HCV
**Behavioral interventions**						
Sacks-Davis et al. (33)	HCV	Narrative synthesis: measures of frequency of injecting risk behaviors varied greatly and could not be pooled	No statistically significant difference in HCV incidence between intervention and control groups (3 studies) The results of the large trial (*n* = 856) tended in the opposite direction to the results in the two smaller trials (*n* = 78 and *n* = 109).	Behavioral interventions are unlikely to have a considerable effect on HCV transmission.	Moderate	Insufficient evidence to support or discount effectiveness of behavioral interventions in prevention HCV.
Hagan et al. (35)	HCV	meta-analysis, random effects model	RR = 1.18, 95% CI: 0.76–1.81 (2 studies)	No evidence that behavioral interventions can have a considerable effect on HCV transmission	Low	
Bhaji et al. (25)	Infectious Endocarditis	Narrative synthesis	Skin and needle hygiene interventional sessions for 6 months, compared to control group: HR = 0.80, 95% CI: 0.37, 1.74 (1 study, *n* = 48).	Tentative evidence to support effectiveness of behavioral interventions to reduce recurrent infectious endocarditis	High	Insufficient SR-level evidence to support effectiveness of educational sessions on skin and needle hygiene in prevention infectious endocarditis (only 1 study)
Addiction services^*^ (including consultation by social work, addiction clinical nurse and/or psychiatry, documentation of addiction in the discharge summary plan, and plan for medication-assisted treatment						
in the community)						
Bahji et al. (25)		Narrative synthesis	50 Patients (49%) were readmitted; 26 (25.5%) died during the study (1 study)	High rates of readmission, re-infection and death in patients who received addiction services; however, the addiction interventions were suboptimal	High	No evidence to support or discount effectiveness of addiction services in prevention of infectious endocarditis
**Interventions targeting population in prison:** Prison based methadone treatment (MMT) program^**^						
Undershill et al. (30) (only one relevant original study included)	HIV	Narrative synthesis; however, only data for 1 study were relevant	1) MMT vs. no MMT aHR = 2.0, 95%CI: 0.9, 4.2 2) MMT for 47-146 days vs. no MMT: aHR = 4.2, 95%CI: 1.4-12.6	1) There were no significant differences in HCV incidence rates between treatment and control groups 2) Short MMT episodes (less than 5 months) were significantly associated with greater risk of HCV	Moderate	Insufficient SR-level evidence to support effectiveness of prison based MMT to prevent HIV (only 1 study)
**Outpatients parenteral antibiotic treatment service**						
Bahji et al. (25)	Infectious endocarditis	Narrative synthesis	Only 2 participants of 28 (7%) had recurrent infectious endocarditis after completing treatment (1 study)	Tentative evidence for effectiveness of outpatients parenteral antibiotic treatment to reduce recurrent infectious endocarditis	High	Insufficient SR-level evidence to support effectiveness of outpatients parenteral antibiotic treatment in prevention of infectious endocarditis

* *No naloxone treatment was received*.

** *Data on discharge policies, adjunctive psychosocial support and urinalysis programmes, which varied between individual prison-based and community based, were not collected*.

## Results

**Figure 1:**
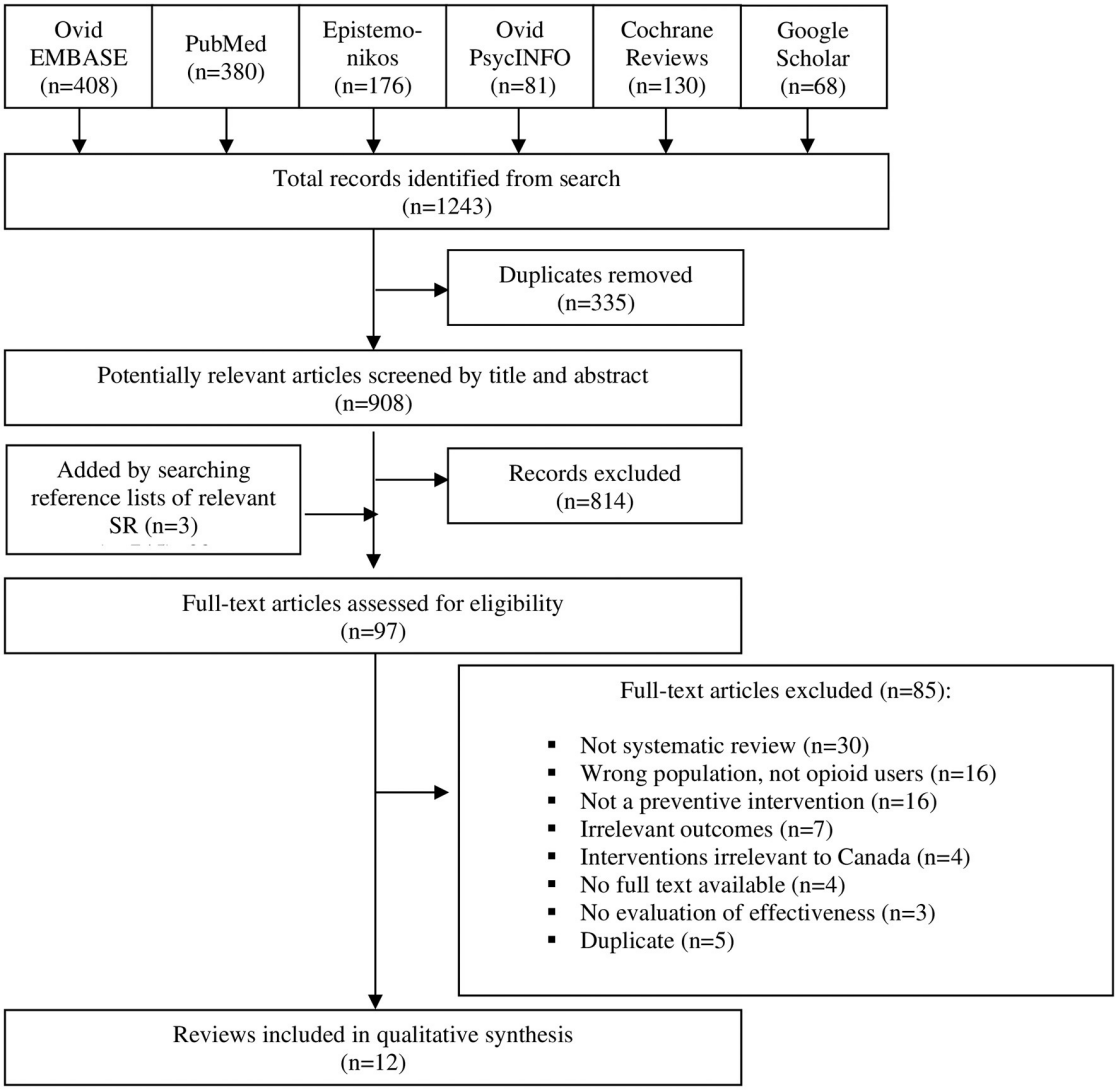
Selection of papers, PRISMA flow diagram.

A total of 1,243 potentially relevant publications were identified via the systematic search (Figure 1). After removing duplicates, 908 potentially relevant citations were screened by title and abstract, and 814 records were excluded. Full texts of the remaining 94 papers and of the additional three papers found by searching bibliographies were screened for eligibility, and 85 papers that were non-relevant or did not meet the eligibility criteria were removed. Twelve SRs were eligible (25–36). Appendix 3 provides a list of the excluded full-text papers with the description of the reasons for exclusion (ordered by most to least common).

Characteristics of included SRs are shown in Tables 3, 4. Of the twelve SRs, eight were identified as core SRs and four as supplemental SRs (see Appendix 5). As reflected in the Citation Matrix (Table 2), among the eligible SRs, twenty primary studies were overlapping and 64 were included only in one SR. In six SRs, the use of opioids was confirmed in all primary studies relevant to our review question (25, 26, 30, 32–36). In the other five SRs (27–29, 31, 35), use of opioids was confirmed in 73–88% of relevant primary studies. In one included SR (26), the use of opioids was confirmed in 33% (two of six) primary studies.

**Figure 2:**
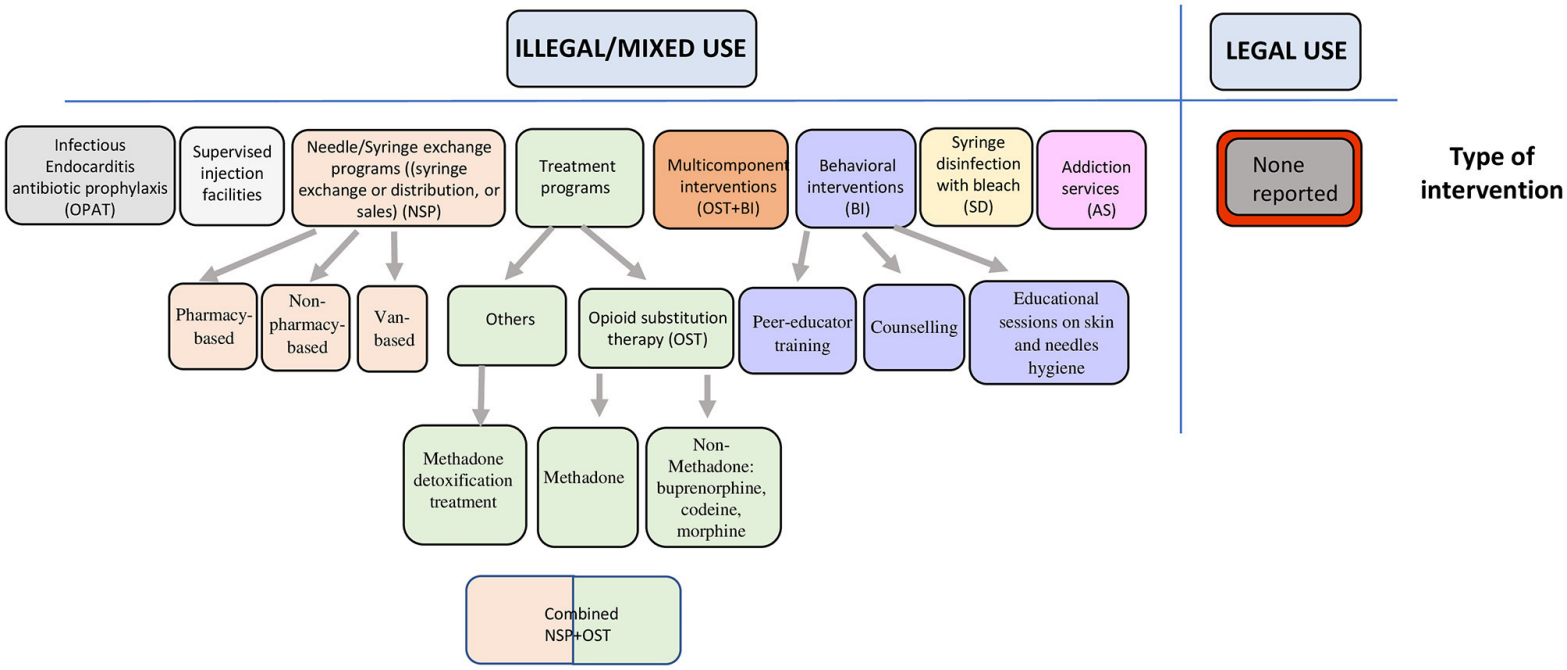
Typology of interventions with reported effectiveness in prevention of common infections in opioid users.

Overall, we identified 8 different types of preventive interventions (Figure 2). Eleven studies (25–36) evaluated opioid substitution therapy (OST) and needle and syringe exchange programs (NSP) to prevent HCV and/or HIV along with several other interventions, and one SR evaluated interventions to prevent infectious endocarditis (25). All included SRs targeted persons who inject drugs, recruited in different settings (Tables 3, 4). Most studies evaluated either HCV (26, 27, 33, 35) or HIV (29, 30, 32, 34) infections prevention, and three studies looked at both. The most common opioid was heroin. One identified SR (38) targeted drug users who were involved with criminal justice (incarcerated, or formerly incarcerated persons).

### Scope of Interventions

A scope of interventions (our first objective) is presented in Figure 2. All SRs evaluated effectiveness of interventions related to the use of non-pharmaceutical opioids. The interventions targeting prevention of viral infections were NSP, OST, combined OST and NSP, syringe disinfection, behavioral interventions, or multi-component interventions. Another group of interventions targeted prevention of infectious endocarditis, which included outpatient parenteral antibiotic treatments and addiction services consultations provided by a social worker, an addiction clinical nurse, or a psychiatrist. Behavioral interventions were educational sessions on skin and needle hygiene.

### Effectiveness of Interventions

**Figure 3:**
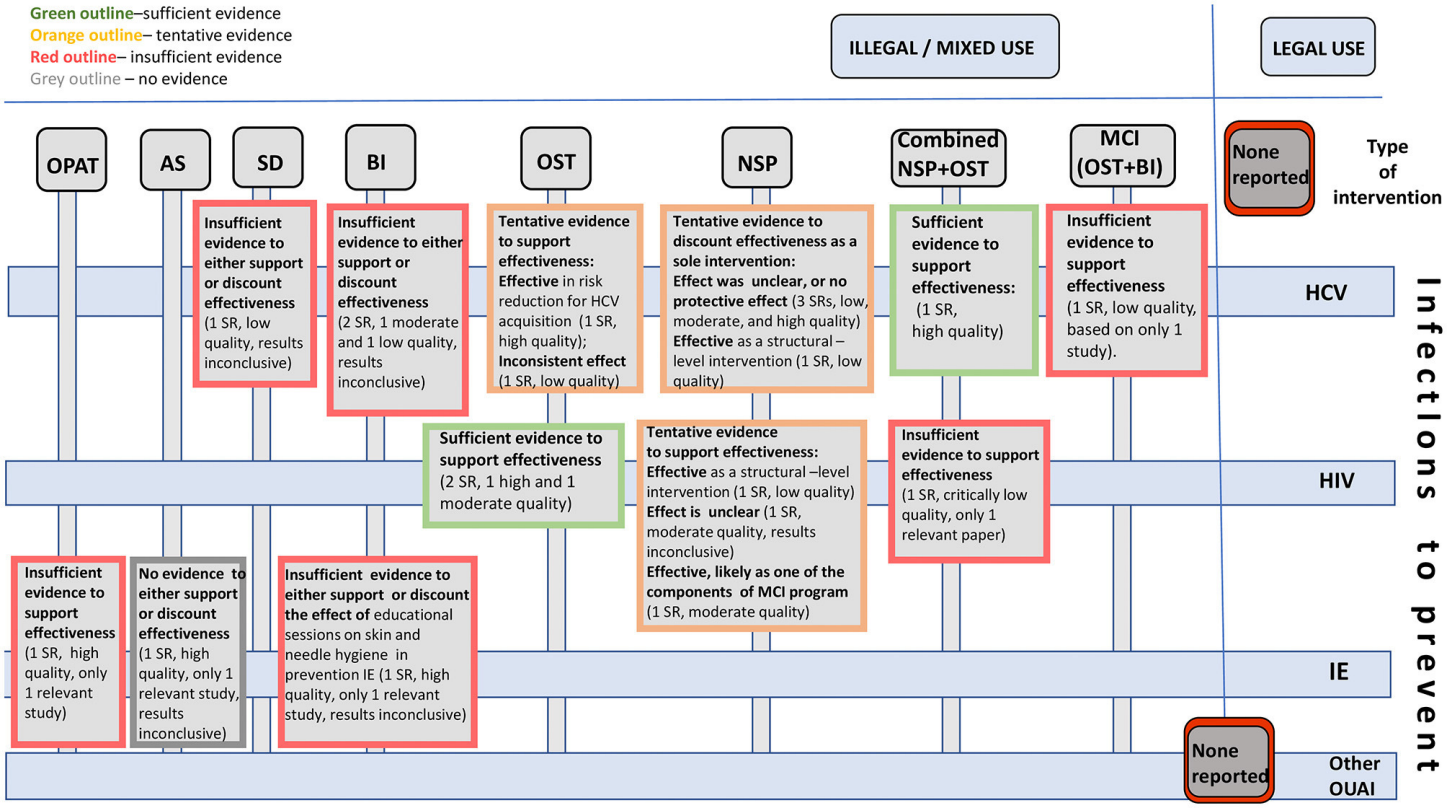
Effectiveness of interventions to prevent infections in opioid users: existing evidence and knowledge gaps.

The results of effectiveness of all interventions are summarized in Tables 5, 6, synthesized in Figure 3, and presented as a Supplementary Figure 1.

#### HCV Prevention

**NSP**. Most included SRs on the use of NSP focused on the prevention of HCV: three SRs studied HCV prevention only and two SRs studied HCV and HIV prevention. One SR (31) (low quality) found that, as a structural-level (public health) intervention, NSP was effective in decreasing HCV prevalence, which was reported by nine primary studies included in the SR. Another SR (28), also devoted to HCV and HIV prevention (moderate quality), stated that pharmacy-based NSP appears to reduce the rate of injection risk behaviors, however, its effect on HCV prevalence remained unclear. The results of the three other SRs (26, 27, 35), high, low, and high quality, respectively, are mixed (Table 1). The pooled effect estimate for ORs (2 primary studies) in the study of Davis et al. (26) suggests protective effect, while the pooled effect estimate for HR (four primary studies studies) implies harmful effect associated with the program (Table 5). However, the wide 95%CI of the pooled RRs and HRs (Table 5) suggest inconclusive evidence for both measures. In the study of Platt et al. (27) (five studies) and Hagan et al. (35) (seven studies), the pooled HR and RR, respectively, and the 95%CI of these effect estimates suggest that PWID who used the program had increased risk for seroconversion compared to those who did not use the NSP. Therefore, we consider that these data provide tentative SR-level evidence to reject effectiveness of NSP as a sole intervention to prevent HCV infection.

**OST** was considered effective in reducing the risk for HCV acquisition in one high quality SR (27). The pooled measure for the relative risk (RR) for HCV incidence in the participants of OST vs. those not participating in OST (twelve primary studies) was 0.50 (95% CI: 0.40, 0.63). In another, low quality, SR (35) the effects of non-specified substance use treatment on HCV seroconversion were considered inconsistent by the authors. However, the pooled RR for opioid replacement therapy was calculated as 0.60 (95% CI: 0.35, 1.03), suggesting protective effect of this program. Considering that the first review was of a high quality and included twelve primary studies and that the results in the SR of Hagan et al. were in favor of the protective effect of OST, we consider this evidence as tentative to support OST effectiveness in preventing HCV.

**Combined OST+NSP** was found to be effective in reducing HCV incidence by one high quality SR (27), with a pooled RR of 0.26 (95% CI: 0.07, 0.79) vs. no OST and no/low NSP. The preventive effect of the combined interventions on HCV infection transmission was also shown in the SR by Hagan et al. (35), even though they studied NSP as a multi-component intervention. Together, these data represent sufficient evidence to support the effectiveness of combined OST and NSP interventions to prevent HCV. Of note, this intervention was also found to be effective (IRR of 0.15, 95% CI: 0.06, 0.40) in one primary study included in the low quality SR (36). Since only one primary study from SR of Jones et al. (36) was relevant to our research question, this was not included in SR of SRs-level synthesis.

**Syringe Disinfection**. Only one low quality SR (35) evaluated the effect of syringe disinfection in prevention of HCV transmission (Table 6). According to the authors (35), this intervention was found to be non-effective. However, the wide 95%CI (pooled RR from four primary studies = 1.08, 95% CI: 0.66, 1.75) suggest that that the results were inconclusive, possibly due to the sparse data. We, therefore, consider this evidence insufficient to support or discount the efficiency of syringe disinfection on HCV transmission.

**Behavioral interventions** were evaluated in two SRs (33, 35); however, two primary studies used in the low quality review of Hagan et al. (35) were overlapping with another, moderate quality review (33). The SR of Sacks-Davis et al. also included three other primary studies. High heterogeneity in study design, outcome measures, and the magnitude, direction, and significance of associations precluded the authors from calculating the pooled effect measures. The trends for the effect of behavioral interventions on HCV incidence observed in one large and two small primary studies had opposite directions, with no significant differences between the intervention and the control groups (Table 6). Our conclusion was that at the SR of SRs-level, the evidence to support or discount the effectiveness of behavioral interventions as a sole intervention to prevent HCV transmission was insufficient.

**Multi-component interventions** in the low quality SR of Hagan et al. (35) were found effective in reducing HCV seroconversion with a pooled RR of 0.25 (0.07, 0.83). This conclusion, however, was based on two primary studies evaluating different combined interventions (Table 6). One of these primary studies (40) evaluated OST combined with enhanced HCV prevention counseling vs. OST alone. Another primary study (41), however, evaluated the combination of OST and NSP. We, therefore, could not consider the evidence from only one study sufficient SR-level evidence to support the effectiveness of OST plus behavioral interventions in HCV prevention.

#### HIV Prevention

**NSP** was found effective to prevent HIV as a public health intervention in one low quality SR (31). A moderate quality SR (28) found the association between NSP and HIV prevalence inconclusive. The authors reported a pooled OR of 0.56 (95% CI: 0.18, 1.77) based on the three primary studies. Another moderate quality SR (29) found a substantial reduction in HIV transmission (RR = 0.42, 95%CI: 0.22, 0.81) across six primary studies involving people who injected opioids. The authors of this SR, however, highlighted that NSPs in these studies may have been combined with other interventions, and thus suggested that NSP be considered as one of the components in a multicomponent program. We, therefore, considered the SR-level evidence of NSP for HIV prevention as tentative.

**OST**. A moderate quality SR (32) and a high quality (34) SR reported OST to be an effective intervention to prevent HIV. In a meta-analysis of MacArthur et al. (32), OST was associated with a protective effect, with a pooled RR of 0.54 (95% CI: 0.32, 0.67), based on nine studies. We considered this as sufficient evidence to support the effectiveness of OST for HIV prevention.

**Opioid maintenance treatment targeting incarcerated, or formerly incarcerated persons** was a methadone-based treatment program evaluated in the moderate quality SR (30). Only one primary study was relevant to our research question. This study suggests an increased risk for HIV seroconversion between participants and non-participants (adjusted HR = 2.0; 95% CI: 0.9, 4.2). However, since these results were based on only one primary study, we considered them as insufficient for SR-level evidence.

**Combined NSP and OST** were found to be effective in reducing HIV incidence by one SR of a critically low quality (36). The results were based on only one relevant primary study; therefore, we do not consider these results as sufficient SR-level evidence.

#### Prevention of Other Infections. Infectious Endocarditis

Only one SR (25) was devoted to interventions to prevent infections other than HIV or HCV, and discussed prevention strategies for recurrent infectious endocarditis in PWID. Most drug users (87%) suffered from an opioid use disorder. The SR included four studies, and three of them had acquisition of infection as an outcome. Each study, however, studied a different intervention (behavioral interventions, outpatient parenteral antibiotic treatment, and addiction services). Therefore, there was no SR-level evidence to support or discount the effectiveness of these interventions in prevention of infectious endocarditis.

### Other Interventions

We did not find any SR evaluating effectiveness of interventions to prevent OUAIs other than those discussed above. We also found no SRs targeting a population of opioid users different from PWID. No SR evaluated legal or mixed opioid use.

## Discussion

This study was designed in response to the marked increase in opioid-related morbidity and mortality during the North American opioid crisis. In our manuscript, we synthesized SR of SRs-level evidence on the scope and effectiveness of interventions for prevention of OUAIs. The estimation of the quality of evidence used for SR data synthesis and for making final conclusions is reflected in the “Conclusion of SR” column. (Tables 5, 6). The conclusions of such synthesis served as the substrates for the SR of SRs level summary and were presented in the Evidence Statement columns (Tables 5, 6). We created a typology of interventions to prevent OUAIs and showed that available SR literature focuses mostly on the interventions to prevent OUAIs in users of non-pharmaceutical opioids, and most participants are PWID. We found SR of SRs-level evidence that supports OST, NSP, and combined interventions in reduction of HCV/HIV transmission in persons who inject opioids. Three serious knowledge gaps in this area were identified: (1) in most SRs, the type of a drug used by the participants was not specified; (2) there was an absence of SRs focusing on the interventions to prevent infections in patients using pharmaceutical opioids prescribed by a health professional; (3) there was a lack of SR-level evidence on OUAIs other than HIV and HCV.

### Effectiveness of Interventions to Prevent OUAIs

There was sufficient and tentative SR-level evidence for the effectiveness of opioid substitution therapy (OST) to prevent HIV and HCV, respectively. Tentative evidence to support effectiveness of needle/syringe exchange programs (NSP) in HIV, but not HCV, prevention, was found. There was sufficient evidence to support the effectiveness of combined OST and NSP interventions in HCV prevention. The SR-level evidence to support the effectiveness of other interventions to prevent OUAIs was insufficient.

Our results are in line with findings of previously published overviews of reviews. More specifically, McArthur et al. (37) found sufficient and tentative SR-level evidence for OST to prevent HIV and HCV, respectively. In terms of NSP intervention, McArthur and colleagues and Palmateer et al. (38) agreed that there was tentative evidence to support the effectiveness of NSP to prevent HIV transmission, and that there was no sufficient evidence to support its effectiveness for the prevention of HCV transmission. In the overview of SRs by Fernandes et al. (42), NSP was found effective in reducing HIV transmission in PWID. The authors also found mixed results on the effectiveness of NSP in HCV prevention and suggested that public health interventions and multi-component programs may be more beneficial. Thus, our results regarding population of persons injecting or co-injecting opioids with other drugs were in line with the results of the overviews from the past, involving PWID who used different kinds of drugs, not necessarily including opioids. SR-level evidence on the effectiveness of interventions to prevent infectious endocarditis remains insufficient.

### Identification of Knowledge Gaps

The third objective of our SR of SRs resulted in the identification of several important knowledge gaps existing in the systematic review literature on the effectiveness of interventions to prevent OUAIs. One of the important omissions in the reviewed literature was not specifying the type of a drug used by the participants, except for the SRs devoted to OST. Furthermore, the co-use of drugs vs. exclusive opioid use was seldom reported. We postulate that this discrepancy could partly explain the mixed results in different primary studies since use of a specific substance vs. co-use of different substances might modify effects on ensuing users' behaviors and its consequences. For example, it has been previously reported (43) that co-use of certain drugs and the number of drugs co-used with pharmaceutical opioids could be an effect modifier in the association between use of opioids and risk for HCV acquisition. The relative excess risk of HCV seroconversion due to interactions was the highest for co-use of injected prescription opioids with injected cocaine, smoked crack/cocaine, and non-injected tranquilisers (43). Therefore, reporting the type of opioid and the co-use of substances is important and needs to be considered when the effectiveness of interventions to prevent infections in opioid users is studied.

Furthermore, we did not find any SR that evaluated interventions to prevent infections in patients using pharmaceutical opioids prescribed by a health professional. These infections, however, despite the legal origin of opioids, can contribute to an increase in opioid-related morbidity and mortality. Adaptive immunity and, therefore, risk to acquire an infection, can depend on the type of opioid (44). For example, morphine, fentanyl and codeine alter/suppress innate and adaptive immunity directly and via the hypothalamic-pituitary axis more than other opioids (45). Hydrocodone, hydromorphone, tramadol, and oxycodone appear to possess low risk in immune system response suppression (45). On the other hand, methadone, although immunosuppressive, might partially restore immune function in heroin users (46–48). These differences are sufficiently substantial to require individual approaches in choosing preventive interventions. This lack of SRs on the interventions to prevent common infections in legal users of pharmaceutical opioids prescribed by a health professional is a serious knowledge gap that needs to be addressed.

Another identified knowledge gap is a lack of SR-level evidence on OUAIs other than HIV and HCV. Most bacterial infections associated with opioid use can result in substantial morbidity. We did find one SR evaluating the effectiveness of interventions to prevent infectious endocarditis (25). However, there were no other studies of sufficiently high quality to support its conclusions. Furthermore, we did not find any SRs devoted to the effectiveness of preventive interventions against other important problems associated with legal and illegal opioid use such as skin infections (soft tissue abscesses, cellulitis), bone infections (osteomyelitis), or fungal infections in persons who inject opioids. None of the SRs studied the effectiveness of interventions to prevent tuberculosis, which is a common problem in street entrenched persons or those living in overcrowded dwellings. It is seen frequently among patients of low socio-economic status and users of nonpharmaceutical opioids. Importantly, we found no SRs looking at the users of pharmaceutical opioids like individuals with chronic pain or populations with multiple co-morbidities. This is a significant gap in today's research as these groups comprise a substantial proportion of legal and mixed opioid users. In addition, no studies focused on different age groups (children, adolescents, elderly etc.). Likewise, current guidelines and recommendations to prevent infections in people with opioid use disorder (49) are focused on HIV and HCV infections prevention, probably due to the lack of studies evaluating prevention of other infections. This knowledge gap was confirmed by our findings.

Our SR of SRs demonstrates that existing evidence on the effectiveness of preventive interventions against HIV and HCV infections is much more abundant than the information on prevention of other OUAIs. This finding suggests that attention should now be re-focused to the less developed knowledge areas. For example, skin and vein care is an essential part of NSP, targeting both viral and bacterial infection complications. There is, however, a lack of studies evaluating effectiveness of NSP, or other interventions, in prevention of skin and vein infections to inform best practices. In addition, the literature suggests that the prevention of sepsis plays an important role in the care of all types of opioid users. In a US cohort based in 373 hospitals, almost half of the mortality outcomes were associated with sepsis diagnosis (50). The proportion of sepsis hospitalizations related to opioid use in this cohort increased by 77% between 2009 and 2015. The authors emphasize an urgent need for the integration of preventive measures such as diagnostic imaging, source control, and empiric antifungal therapy to decrease the risk of infectious complications.

Given the ubiquitous legal and illegal use of opioids in North America, including long-term use in high immunosuppressive doses, the knowledge on the effective prevention of OUAIs is of paramount importance to all healthcare professionals encountering these vulnerable patients as well as to the policy and decision makers.

## Strength and Limitations

To our knowledge, this is the first broad scope SR of SRs that synthesized SR-level evidence on the effectiveness of interventions to prevent common infections in people who use pharmaceutical and non-pharmaceutical opioids, legally and/or illegally, via any route of administration. It was conducted in response to the North American opioid crisis and in an effort to help curb opioid-related morbidity and mortality. We have followed a pre-specified, registered protocol. The quality assessment of included SRs was conducted, and the quality of synthesized evidence was graded using previously used validated tools. The main strength of the present work is its SR of SRs design. This methodology allows synthesizing the highest level of evidence in a user-friendly format to help knowledge users and policy makers to take informed decisions.

There are some potential limitations. First, this study is limited to the evidence from published SRs only. Thus, we exclude the evidence from other types of literature. Moreover, most eligible SRs were focused on population-based interventions, therefore, the conclusions made by the authors of these SRs should be considered with respect to the limitations of observational studies. Further, our definition of SR complies with the PRISMA checklist. Therefore, some studies published as SRs that did not meet the PRISMA checklist selection criteria were excluded. This approach may have restricted the scope of interventions. Second, there could have been a language bias for publications in languages other than English and French. Our intention, however, was to synthesize the evidence on interventions most relevant to the North American context. Finally, we could not use the GRADE system to evaluate the level of certainty of the evidence since it has not yet been developed for SR of SRs. We, however, used a method that has been previously applied to the published overview of reviews.

## Conclusion

Our SR of SRs demonstrates that current focus of existing SRs evaluating effectiveness of interventions to prevent opioid use-associated infections centers almost exclusively on interventions to prevent HCV and HIV transmission and acquisition in users of non-pharmaceutical opioids, specifically in PWID. Of all interventions, the SR of SRs-level of evidence was the strongest for the effectiveness of OST in prevention of HIV (sufficient) and HCV (tentative), for the effectiveness of NSP to prevent HIV (tentative), and for the effectiveness of the combination of both these interventions to prevent HCV (sufficient). The evidence on prevention of recurrent infectious endocarditis in persons who inject opioids is scarce and does not allow for an SR-level conclusion. We identified several important knowledge gaps, such as a scarcity of SR-level evidence on the interventions to prevent infections other than HCV and HIV, as well as intervention targeting users of pharmaceutical opioids in legal and mixed opioid users. Systematic implementation of interventions with known effectiveness will assist in curbing opioid-related morbidity and mortality. Knowledge gaps identified in our study should be addressed by researchers and policy makers.

## Data Availability Statement

The original contributions presented in the study are included in the article/Supplementary Material, further inquiries can be directed to the corresponding author/s.

## Author Contributions

SP: conceptualization, methodology, formal analysis, data curation, visualization, writing—original draft, and writing—review and editing. ME: conceptualization, supervision, and writing—review and editing. KF, AH-L, MM, and SW: writing—review and editing. GG: methodology and writing—review and editing. EP: conceptualization, data curation, and writing—review and editing. IK: conceptualization, methodology, formal analysis, supervision, data curation, and writing—review and editing. All authors contributed to the article and approved the submitted version.

## Funding

This study was funded by the Canadian Institutes of Health Research (#EOC-162067). ME was the Principal Investigator on this grant. IK was supported by la Lettre d'entente no 250 (chercheurs en médecine de famille, from the Ministère de la Santé et des Services sociaux du Québec - Fédération des médecins omnipraticiens du Québec/Fonds de recherche du Québec – Santé). KF was supported by a Senior Research Scholar award from the Fonds de recherche du Québec – Santé and a William Dawson Scholar award from McGill University.

## Conflict of Interest

The authors declare that the research was conducted in the absence of any commercial or financial relationships that could be construed as a potential conflict of interest.

## Publisher's Note

All claims expressed in this article are solely those of the authors and do not necessarily represent those of their affiliated organizations, or those of the publisher, the editors and the reviewers. Any product that may be evaluated in this article, or claim that may be made by its manufacturer, is not guaranteed or endorsed by the publisher.

## Supplementary Material

Appendix 1 List of SRs identified by the overarching study.

Appendix 2 PRISMA checklist.

Appendix 3 List of excluded SR after full-text screening and reasons for exclusion (excel document).

Appendix 4 Search strategy.

Appendix 5 Methods, detailed.

Supplementary Figure 1 Graphical abstract: Effectiveness of interventions to prevent infections in persons who use opioids: existing evidence and knowledge gaps.

## Abbreviations

OUAIs: Opioid use-associated infections
SR: Systematic review
SR of SRs: Systematic review of Systematic reviews
HCV: Hepatitis C virus
HIV: Human Immunodeficiency virus
PWID: Persons who inject drugs
AMSTAR2: A measurement tool to assess systematic reviews
OPAT: Outpatients parenteral antibiotic treatment
NSP: Needle and syringe exchange programs
NEP: Needle exchange programs
OST: Opioid substitution treatment programs
MMT: Methadone maintenance treatment
MD: Methadone detoxication treatment
LAAM: Levacetylmethadol.
