# Supplementary material for: Effectiveness of Interventions for Prevention of Common Infections Among Opioid Users: A Systematic Review of Systematic Reviews
Source: Front Public Health. 2022 Feb 22;10:749033. doi: 10.3389/fpubh.2022.749033 (PMC8901608; doi:10.3389/fpubh.2022.749033)
Supplement: Appendix 1 — List of SRs identified by the overarching study. [file Data_Sheet_1.pdf]

Appendix 1

List of the systematic reviews related to OUAIs prevention identified by the search for the overarching research project (Canadian Institutes of Health Research [CIHR] grant #EOC-162067) performed in 2019.

1. Davis SM, Daily S, Kristjansson AL, et al. Needle exchange programs for the prevention of hepatitis C virus infection in people who inject drugs: A systematic review with meta-analysis. *Harm Reduction Journal Vol 14 2017, ArtID 25.* 2017;14.
2. Gowing L, Farrell MF, Bornemann R, Sullivan LE, Ali R. Oral substitution treatment of injecting opioid users for prevention of HIV infection. *Cochrane Database Syst Rev.* 2011(8):Cd004145.
3. Guise A, Seguin M, Mburu G, et al. Integrated opioid substitution therapy and HIV care: A qualitative systematic review and synthesis of client and provider experiences. *AIDS Care.* 2017;29(9):1119-1128.
4. Jarlais DCD. Systematic review research on needle/syringe programs and opiate substitution programs in low- and middle-income countries. *Journal of Food and Drug Analysis.* 2013;21(4):S59-S61.
5. Jones L, Pickering L, Sumnall H, McVeigh J, Bellis MA. Optimal provision of needle and syringe programmes for injecting drug users: A systematic review. *Int J Drug Policy.* 2010;21(5):335-342.
6. MacArthur GJ, Minozzi S, Martin N, et al. Opiate substitution treatment and HIV transmission in people who inject drugs: Systematic review and meta-analysis. *BMJ: British Medical Journal.* 2012;345(7879):1-16.
7. Platt L, Minozzi S, Reed J, et al. Needle syringe programmes and opioid substitution therapy for preventing hepatitis C transmission in people who inject drugs. *Cochrane Database Syst Rev.* 2017;9:Cd012021.
8. Prendergast ML, Urada D, Podus D. Meta-analysis of HIV risk-reduction interventions within drug abuse treatment programs. *Journal of Consulting and Clinical Psychology.* 2001;69(3):389-405.
9. Sacks-Davis R, Horyniak D, Grebely J, Hellard M. Behavioural interventions for preventing hepatitis C infection in people who inject drugs: A global systematic review. *International Journal of Drug Policy.* 2012;23(3):176-184.
10. Vold JH, Aas C, Leiva RA, et al. Integrated care of severe infectious diseases to people with substance use disorders; a systematic review. *BMC Infect Dis.* 2019;19(1):306.
11. Weissman J, Kanamori M, Devieux JG, Trepka MJ, De La Rosa M. HIV risk reduction interventions among substance-abusing reproductive-age women: A systematic review. *AIDS Education and Prevention.* 2017;29(2):121-140.
12. Wright NM, Tompkins CN. A review of the evidence for the effectiveness of primary prevention interventions for hepatitis C among injecting drug users. *Harm reduction journal.* 2006;3:27.
13. Haldane V, Cervero-Liceras F, Chuah FL, et al. Integrating HIV and substance use services: a systematic review. *Journal of the International AIDS Society.* 2017;20(1):21585.
14. Oldfield BJ, Muñoz N, McGovern MP, et al. Integration of care for HIV and opioid use disorder. *Aids.* 2019;33(5):873-884.
15. Meader N, Li R, Des Jarlais DC, Pilling S. Psychosocial interventions for reducing injection and sexual risk behaviour for preventing HIV in drug users. *Cochrane Database of Systematic Reviews.* 2010(1).

16. Low AJ, Mburu G, Welton NJ, et al. Impact of opioid substitution therapy on antiretroviral therapy outcomes: a systematic review and meta-analysis. *Clinical Infectious Diseases.* 2016;63(8):1094-1104.
17. Hajarizadeh B, Cunningham EB, Reid H, Law M, Dore GJ, Grebely J. Direct-acting antiviral treatment for hepatitis C among people who use or inject drugs: a systematic review and meta-analysis. *The lancet Gastroenterology & hepatology.* 2018;3(11):754-767.
18. Walsh N, Verster A, Rodolph M, Akl EA. WHO guidance on the prevention of viral hepatitis B and C among people who inject drugs. *International Journal of Drug Policy.* 2014;25(3):363-371.

Appendix 2

PRISMA 2020 Checklist

Section and Topic	Item #	Checklist item	Location where item is reported
**TITLE**			
Title	1	Identify the report as a systematic review.	
**ABSTRACT**			
Abstract	2	See the PRISMA 2020 for Abstracts checklist.	
**INTRODUCTION**			
Rationale	3	Describe the rationale for the review in the context of existing knowledge.	
Objectives	4	Provide an explicit statement of the objective(s) or question(s) the review addresses.	
**METHODS**			
Eligibility criteria	5	Specify the inclusion and exclusion criteria for the review and how studies were grouped for the syntheses.	
Information sources	6	Specify all databases, registers, websites, organisations, reference lists and other sources searched or consulted to identify studies. Specify the date when each source was last searched or consulted.	
Search strategy	7	Present the full search strategies for all databases, registers and websites, including any filters and limits used.	
Selection process	8	Specify the methods used to decide whether a study met the inclusion criteria of the review, including how many reviewers screened each record and each report retrieved, whether they worked independently, and if applicable, details of automation tools used in the process.	
Data collection process	9	Specify the methods used to collect data from reports, including how many reviewers collected data from each report, whether they worked independently, any processes for obtaining or confirming data from study investigators, and if applicable, details of automation tools used in the process.	
Data items	10a	List and define all outcomes for which data were sought. Specify whether all results that were compatible with each outcome domain in each study were sought (e.g. for all measures, time points, analyses), and if not, the methods used to decide which results to collect.	
	10b	List and define all other variables for which data were sought (e.g. participant and intervention characteristics, funding sources). Describe any assumptions made about any missing or unclear information.	
Study risk of bias assessment	11	Specify the methods used to assess risk of bias in the included studies, including details of the tool(s) used, how many reviewers assessed each study and whether they worked independently, and if applicable, details of automation tools used in the process.	
Effect measures	12	Specify for each outcome the effect measure(s) (e.g. risk ratio, mean difference) used in the synthesis or presentation of results.	
Synthesis methods	13a	Describe the processes used to decide which studies were eligible for each synthesis (e.g. tabulating the study intervention characteristics and comparing against the planned groups for each synthesis (item #5)).	
	13b	Describe any methods required to prepare the data for presentation or synthesis, such as handling of missing summary statistics, or data conversions.	
	13c	Describe any methods used to tabulate or visually display results of individual studies and syntheses.	
	13d	Describe any methods used to synthesize results and provide a rationale for the choice(s). If meta-analysis was performed, describe the model(s), method(s) to identify the presence and extent of statistical heterogeneity, and software package(s) used.	
	13e	Describe any methods used to explore possible causes of heterogeneity among study results (e.g. subgroup analysis, meta-regression).	
	13f	Describe any sensitivity analyses conducted to assess robustness of the synthesized results.	
Reporting bias assessment	14	Describe any methods used to assess risk of bias due to missing results in a synthesis (arising from reporting biases).	
Certainty assessment	15	Describe any methods used to assess certainty (or confidence) in the body of evidence for an outcome.	

PRISMA 2020 Checklist

Section and Topic	Item #	Checklist item	Location where item is reported
**RESULTS**			
Study selection	16a	Describe the results of the search and selection process, from the number of records identified in the search to the number of studies included in the review, ideally using a flow diagram.	
	16b	Cite studies that might appear to meet the inclusion criteria, but which were excluded, and explain why they were excluded.	
Study characteristics	17	Cite each included study and present its characteristics.	
Risk of bias in studies	18	Present assessments of risk of bias for each included study.	
Results of individual studies	19	For all outcomes, present, for each study: (a) summary statistics for each group (where appropriate) and (b) an effect estimate and its precision (e.g. confidence/credible interval), ideally using structured tables or plots.	
Results of syntheses	20a	For each synthesis, briefly summarise the characteristics and risk of bias among contributing studies.	
	20b	Present results of all statistical syntheses conducted. If meta-analysis was done, present for each the summary estimate and its precision (e.g. confidence/credible interval) and measures of statistical heterogeneity. If comparing groups, describe the direction of the effect.	
	20c	Present results of all investigations of possible causes of heterogeneity among study results.	
	20d	Present results of all sensitivity analyses conducted to assess the robustness of the synthesized results.	
Reporting biases	21	Present assessments of risk of bias due to missing results (arising from reporting biases) for each synthesis assessed.	
Certainty of evidence	22	Present assessments of certainty (or confidence) in the body of evidence for each outcome assessed.	
**DISCUSSION**			
Discussion	23a	Provide a general interpretation of the results in the context of other evidence.	
	23b	Discuss any limitations of the evidence included in the review.	
	23c	Discuss any limitations of the review processes used.	
	23d	Discuss implications of the results for practice, policy, and future research.	
**OTHER INFORMATION**			
Registration and protocol	24a	Provide registration information for the review, including register name and registration number, or state that the review was not registered.	
	24b	Indicate where the review protocol can be accessed, or state that a protocol was not prepared.	
	24c	Describe and explain any amendments to information provided at registration or in the protocol.	
Support	25	Describe sources of financial or non-financial support for the review, and the role of the funders or sponsors in the review.	
Competing interests	26	Declare any competing interests of review authors.	
Availability of data, code and other materials	27	Report which of the following are publicly available and where they can be found: template data collection forms; data extracted from included studies; data used for all analyses; analytic code; any other materials used in the review.	

From: Page MJ, McKenzie JE, Bossuyt PM, Boutron I, Hoffmann TC, Mulrow CD, et al. The PRISMA 2020 statement: an updated guideline for reporting systematic reviews. BMJ 2021;372:n71. doi: 10.1136/bmj.n71

For more information, visit: http://www.prisma-statement.org/

Appendix 3.

List of studies excluded after full text screening

Abbreviations. SR: Systematic Review; FT: Full Text.

Year	Authors	Title	Reasons for exclusion
2013	Alavi, S. M. and Sarmast Shushtari, M. H.	HIV/AIDS among injecting drug users: A review on epidemiology and management of	Not a SR
2017	Bajis, Sahar and Dore, Gregory J. and Hajarizadeh, Behzad and Cunningham, Evan B.	Interventions to enhance testing, linkage to care and treatment uptake for hepatitis C virus	Not a preventive intervention
2019	Baker, P. and Beletsky, L. and Avalos, L. and Venegas, C. and Strathdee, S. A. and	Policing as a structural determinant of HIV risk among people who inject drugs: A systematic	No FT
2014	Baral, Stefan and Holland, Claire E. and Shannon, Kate and Logie, Carmen and	Enhancing benefits or increasing harms: Community responses for HIV Among men who have	Not a SR
2019	Bazzi, A. R. and Drainoni, M. L. and Biancarelli, D. L. and Hartman, J. J. and Mimiaga,	Systematic review of HIV treatment adherence research among people who inject drugs in the	Not a preventive intervention
2017	Bernard, C. L. and Owens, D. K. and Goldhaber-Fiebert, J. D. and Brandeau, M. L.	Estimation of the cost-effectiveness of HIV prevention portfolios for people who inject drugs in the	Not a SR
2013	Burnhams, Nadine Harker and Musekiwa, Alfred and Parry, Charles and London, Leslie	A systematic review of evidence-based workplace prevention programmes that address	Wrong population
2019	Chou, R. and Evans, C. and Hoverman, A. and Sun, C. and Dana, T. and Bougatsos,	Preexposure prophylaxis for the prevention of HIV infection: Evidence report and systematic	Wrong population
2015	Coats, Josh T and Dillon, John F	The effect of introducing point-of-care or dried blood spot analysis on the uptake of hepatitis C	No evaluation of effectiveness
2006	Copenhaver, Michael M. and Johnson, Blair T. and Lee, I. Ching and Harman, Jennifer	Behavioral HIV risk reduction among people who inject drugs: Meta-analytic evidence of efficacy	Wrong outcome
2019	Cortesi, P. A. and Barca, R. and Giudicatti, G. and Mossini, S. and Ciaccio, A. and	Systematic review: economic evaluations of HCV screening in the direct-acting antivirals era	Not a preventive intervention
2014	Craig, A. P. and Thein, H. H. and Zhang, L. and Gray, R. T. and Henderson, K. and	Spending of HIV resources in Asia and Eastern Europe: systematic review reveals the need to	Wrong population
2010	L Degenhardt, B Mathers, P Vickerman, T Rhodes et al.	Prevention of HIV infection for people who inject drugs: why individual, structural, and combination	Not an SR
2010	Degenhardt, Louisa and Mathers, Bradley and Vickerman, Peter and Rhodes, Tim and	Prevention of HIV infection for people who inject drugs: why individual, structural, and	Duplicate
2014	Degenhardt, Louisa and Mathers, Bradley M and Wirtz, Andrea L and Wolfe, Daniel	What has been achieved in HIV prevention, treatment and care for people who inject drugs,	Wrong outcome
2013	Des Jarlais, D. C. and Feelemyer, J. P. and Modi, S. N. and Abdul-Quader, A. and	High coverage needle/syringe programs for people who inject drugs in low and middle income	not relevant to Canada
2017	Fernandes, R. M. and Cary, M. and Duarte, G. and Jesus, G. and Alarcao, J. and	Effectiveness of needle and syringe Programmes in people who inject drugs - An overview of	Not a SR
2017	Ghosh, Debarchana and Krishnan, Archana and Gibson, Britton and Brown, Shan-	Social network strategies to address HIV prevention and treatment continuum of care among at-	Not a SR
1999	Gibson, D. R. and Flynn, N. M. and McCarthy, J. J.	Effectiveness of methadone treatment in reducing HIV risk behavior and HIV seroconversion	Not a SR
1998	Gibson, D. R. and McCusker, J. and Chesney, M.	Effectiveness of psychosocial interventions in preventing HIV risk behaviour in injecting drug	Not a SR
2001	Gibson, David R., Flynn, Neil M., Perales, Daniel	Effectiveness of syringe exchange programs in reducing HIV risk	Not an SR
2017	Gilchrist, G. and Swan, D. and Widyaratna, K. and Marquez-Arrico, J. E. and Hughes,	A Systematic Review and Meta-analysis of Psychosocial Interventions to Reduce Drug and	Wrong ouotcome
2010	Gillies, M. and Palmateer, N. and Hutchinson, S. and Ahmed, S. and Taylor, A. and	The provision of non-needle/syringe drug injecting paraphernalia in the primary prevention of HCV	Wrong outcome
2013	Gomez, G. B. and Borquez, A. and Case, K. K. and Wheelock, A. and Vassall, A. and	The cost and impact of scaling up pre-exposure prophylaxis for HIV prevention: a systematic	Wrong population
2008	Gowing, L. and Farrell, M. and Bornemann, R. and Ali, R.	Substitution treatment of injecting opioid users for prevention of HIV infection	No FT
2017	Guise, Andy and Seguin, Maureen and Mburu, Gitau and McLean, Susie and Grenfell,	Integrated opioid substitution therapy and HIV care: A qualitative systematic review and synthesis	No evaluation of effectiveness
2013	Hahne, S. J. and Veldhuijzen, I. K. and Wiessing, L. and Lim, T. A. and Salminen, M.	Infection with hepatitis B and C virus in Europe: a systematic review of prevalence and cost-	Not a preventive intervention
2013	Jarlais, D. C. D.	Systematic review research on needle/syringe programs and opiate substitution programs in low-	Not relevant to Canada
2014	Jiwatram-Negron, Tina and El-Bassel, Nabila	Systematic review of couple-based HIV intervention and prevention studies: Advantages, gaps,	Wrong population
2012	John-Baptiste, A. and Yeung, M. W. and Leung, V. and van der Velde, G. and Krahn,	Cost effectiveness of hepatitis C-related interventions targeting substance users and other high-	Wrong population
2014	Jones, Lisa and Bates, Geoff and McCoy, Ellie and Beynon, Caryl and McVeigh, James	Effectiveness of interventions to increase hepatitis C testing uptake among high-risk groups: A	Wrong population
2014	L. Jones, G. Bates, and E. McCoy, and C. Beynon, and J. McVeigh, MA Bellis	Effectiveness of interventions to increase hepatitis C testing uptake among high-risk groups: A	Duplicate
2018	Kalafateli, M. and Buzzetti, E. and Thorburn, D. and Davidson, B. R. and Tsochatzis, E.	Pharmacological interventions for acute hepatitis C infection	Wrong population
2018	Lazarus, J. V. and Safreed-Harmon, K. and Hetherington, K. L. and Bromberg, D. J.	Health Outcomes for Clients of Needle and Syringe Programs in Prisons	Wrong population
2011	Linda, Gowing and Michael, F. Farrell and Reinhard, Bornemann and Lynn, E. Sullivan	Oral substitution treatment of injecting opioid users for prevention of HIV infection	Duplicate:
2002	Lucidarme, D.	[Hepatitis C and drug use: epidemiology, screening, natural history and treatment]	Not a SR
2014	MacArthur, Georgina J and van Velzen, Eva and Palmateer, Norah and Kimber, Jo and	Interventions to prevent HIV and hepatitis C in people who inject drugs: a review of reviews to	Not a SR
2020	Magwood, Olivia and Salvalaggio, Ginetta and Beder, Michaela and Kendall, Claire and	The effectiveness of substance use interventions for homeless and vulnerably housed persons: A	Not a preventive intervention
2017	Mantzoukis, K. and RodrÃ-gueză€ PerÃ¡lvarez, M. and Buzzetti, E. and Thorburn, D.	Pharmacological interventions for acute hepatitis B infection	Not a preventive intervention
2014	Marrazzo, J. M. and del Rio, C. and Holtgrave, D. R. and Cohen, M. S. and Kalichman,	HIV prevention in clinical care settings: 2014 recommendations of the International Antiviral	Not a SR
2019	Marshall, L. and Teljeur, C. and Murchu, E. O. and Harrington, P. and Ryan, M.	Pin125 Systematic Review of Cost-Effectiveness: Pre-Exposure Prophylaxis (Prep) for the	no FT
2013	Martin, N. K. and Hickman, M. and Miners, A. and Hutchinson, S. J. and Taylor, A. and	Cost-effectiveness of increasing HCV case-finding for people who inject drugs VIA dried blood	Not a SR
2000	Merson, M. H. and Dayton, J. M. and O'Reilly, K.	Effectiveness of HIV prevention interventions in developing countries	no FT
2019	Murdock, Rachel M and Brizzi, Marisa B and Perez, Omar and Badowski, Melissa E	Public health considerations among people who inject drugs with HIV/HCV co-infection: a review	Not a SR
2013	Noel, Lina and Gagnon, Dominique	Systematic review of peer-based interventions with injection drug users	Wrong outcomes
2010	Palmateer, Norah and Kimber, Jo and Hickman, Matthew and Hutchinson, Sharon and	Evidence for the effectiveness of sterile injecting equipment provision in preventing hepatitis C	Not a SR
2013	Palmateer, N. E. and Hutchinson, S. J. and Innes, H. and Schnier, C. and Wu, O. and	Review and meta-analysis of the association between self-reported sharing of needles/syringes	Not a preventive intervention
2018	Paquette, Catherine E. and Pollini, Robin A.	Injection drug use, HIV/HCV, and related services in nonurban areas of the United States: A	No evaluation of effectiveness
2019	Pitcher, A. B. and Borquez, A. and Skaathun, B. and Martin, N. K.	Mathematical modeling of hepatitis c virus (HCV) prevention among people who inject drugs: A	Not a SR
2005	Pitisuttithum, P.	HIV-1 prophylactic vaccine trials in Thailand	Not a SR
2018	Platt, L. and Minozzi, S. and Reed, J. and Vickerman, P. and Hagan, H. and French, C.	Needle and syringe programmes and opioid substitution therapy for preventing HCV transmission	Duplicate
2016	Platt, Lucy and Reed, Jennifer and Minozzi, Silvia and Vickerman, Peter and Hagan,	Effectiveness of needle/syringe programmes and opiate substitution therapy in preventing HCV	Not a SR
2001	Prendergast, Michael L. and Urada, Darren and Podus, Deborah	Meta-analysis of HIV risk-reduction interventions within drug abuse treatment programs	Wrong outcome
2019	Radley, A. and Robinson, E. and Aspinall, E. J. and Angus, K. and Tan, L. and Dillon,	A systematic review and meta-analysis of community and primary-care-based hepatitis C testing	Wrong population
2019	Reddon, Hudson and Marshall, Brandon DL and Milloy, MJ	Elimination of HIV transmission through novel and established prevention strategies among	Not a SR
2018	Restar, A. and Nguyen, M. and Nguyen, K. and Adia, A. and Nazareno, J. and	Trends and emerging directions in HIV risk and prevention research in the Philippines: A	Wrong population
2006	Ritter, A. and Cameron, J.	A review of the efficacy and effectiveness of harm reduction strategies for alcohol, tobacco and	Not a SR effect estimate but veru useful for discussion
2019	RodrÃ-guez-SÃ¡nchez, B. and Cercenado, E. and Coste, A. T. and Greub, G.	Review of the impact of MALDI-TOF MS in public health and hospital hygiene, 2018	Wrong population
2012	Ruger, Jennifer Prah and Lazar, Christina M.	Economic evaluation of drug abuse treatment and HIV prevention programs in pregnant women:	Not a SR
2007	Scheinmann, R. and Hagan, H. and Lelutiu-Weinberger, C. and Stern, R. and Des	Non-injection drug use and Hepatitis C Virus: a systematic review	Not a preventive intervention
2017	Simeone, Claire A. and Seal, Stella M. and Savage, Christine	Implementing HIV testing in substance use treatment programs: A systematic review	Not a preventive intervention
2000	James L. Sorensen , Amy L. Copeland	Drug abuse treatment as an HIV prevention strategy: a review	Not a SR
2013	Suthar, A. B. and Ford, N. and Bachanas, P. J. and Wong, V. J. and Rajan, J. S. and	Towards universal voluntary HIV testing and counselling: a systematic review and meta-analysis	Not a preventive intervention
2007	Tilson, Hugh and Aramrattana, Apinun and Bozzette, Samuel and Celentano, David	Preventing HIV infection among injecting drug users in high-risk countries: an assessment of the	Non-relevant to Canada
2019	Tressler, S. and Bhandari, R.	Interventions to increase completion of hepatitis B vaccination in people who inject drugs: A	Not a preventive intervention
2011	Turner, Katy ME and Hutchinson, Sharon and Vickerman, Peter and Hope, Vivian and	The impact of needle and syringe provision and opiate substitution therapy on the incidence of	Not a SR
2014	Uuskula, A. and Raag, M. and Folch, C. and Prasad, L. and Karnite, A. and van Veen,	Self-reported testing, HIV status and associated risk behaviours among people who inject drugs in	Not a SR
2020	Van Remoortel, H. and Matthysen, W. and Avau, B. and Compernolle, V. and	Is sexual risk behaviour associated with an increased risk of transfusion-transmissible infections	Not a preventive intervention
2010	Vlahov, David and Robertson, Angela M and Strathdee, Steffanie A	Prevention of HIV infection among injection drug users in resource-limited settings	Not a SR
2019	Vold, J. H. and Aas, C. and Leiva, R. A. and Vickerman, P. and Chalabianloo, F. and	Integrated care of severe infectious diseases to people with substance use disorders; a	Wrong population
2019	Wakeman, Sarah E	Harm Reduction Approaches for Opioid Use Disorder	Not a SR
2017	Weissman, J. and Kanamori, M. and Devieux, J. G. and Trepka, M. J. and De La Rosa,	HIV risk reduction interventions among substance-abusing reproductive-age women: A systematic	Not a preventive intervention
2017	Wiessing, L. and Ferri, M. and Belackova, V. and Carrieri, P. and Friedman, S. R. and	Monitoring quality and coverage of harm reduction services for people who use drugs: a	Not a SR
2014	Wiessing, L. and Ferri, M. and Grady, B. and Kantzanou, M. and Sperle, I. and Cullen,	Hepatitis C virus infection epidemiology among people who inject drugs in Europe: a systematic	Not a preventive intervention
2004	A. Woodak and A. Cooney	Effectiveness of sterile needle and syringe programming in reducing HIV/AIDS among injecting dru	Not an SR
2006	Wright, N. M. and Tompkins, C. N.	A review of the evidence for the effectiveness of primary prevention interventions for hepatitis C	Not a SR
2020	Yanes-Lane, M. and Dussault, C. and Linthwaite, B. and Cox, J. and Klein, M. B. and	Using the barriers and facilitators to linkage to HIV care to inform hepatitis C virus (HCV) linkage	Wrong population
2019	Yen-Hao Chu, I. and Wen-Wei Ku, S. and Li, C. W. and Toh, H. S. and Yang, C. J. and	Taiwan guideline on oral pre-exposure prophylaxis for HIV prevention - 2018 update	Not a SR
2007	Young, T. N. and Arens, F. J. and Kennedy, G. E. and Laurie, J. W. and Rutherford, G.	Antiretroviral postâ€ exposure prophylaxis (PEP) for occupational HIV exposure	Wrong population
2009	Zanini, B. and Lanzini, A.	Antiviral treatment for chronic hepatitis C in illicit drug users: A systematic review	Wrong population
2019	Zhang, Chen and McMahon, James and Simmons, Janie and Brown, L Lauren and	Suboptimal HIV Pre-exposure prophylaxis awareness and willingness to use among women who	Wrong outcome
2019	Zhou, B. and Cai, G. F. and Lv, H. K. and Xu, S. F. and Wang, Z. T. and Jiang, Z. G.	Factors correlating to the development of hepatitis c virus infection among drug users-findings	Not a preventive intervention
2019	Zhou, B. and Cai, G. F. F. and Lv, H. K. K. and Xu, S. F. F. and Wang, Z. T. T. and	Systematic Review and Meta-Analysis	Duplicate
2012	Zhuang, X. and Liang, Y. and Chow, E. P. F. and Wang, Y. and Wilson, D. P. and	HIV and HCV prevalence among entrants to methadone maintenance treatment clinics in China: a	Not a preventive intervention
2012	Zhuang, Xun and Wang, Yafei and Chow, Eric P. and Liang, Yanxian and Wilson,	Risk factors associated with HIV/HCV infection among entrants in methadone maintenance	Non-relevant to Canada

Appendix 4.

Search strategy.

Search strategy, PubMed

Concept #1, Opioids:

("analgesics, opioid"[mh] OR "analgesics, opioid"[pa] OR "Controlled Substances" [majr] OR narcotics[mh:noexp] OR "Opioid-Related Disorders" [mh] OR narcotic*[tw] OR opiate*[tw] OR opioid*[tw] OR acetylmethadol[tw] OR alfentanil[tw] OR anileridine[tw] OR Benzomorphan*[tw] OR bezitramide[tw] OR buprenorphine[tw] OR butorphanol[tw] OR Codeine[tw] OR Dextromethorphan[tw] OR Dextromoramide[tw] OR Dextropropoxyphene[tw] OR dezocine[tw] OR Diamorphine[tw] OR dihydrocodein*[tw] OR Diphenylpropylamine[tw] OR Ethylmorphine[tw] OR Fentanyl*[tw] OR Heroin[tw] OR Hydrocodon*[tw] OR Hydromorphon*[tw] OR ketobemidone[tw] OR levacetylmethadol[tw] OR Meperidine[tw] OR Meptazinol[tw] OR methadone[tw] OR Morphan*[tw] OR Morphin*[tw] OR nalbuphine[tw] OR nicomorphine[tw] OR normethadone[tw] OR Opium[tw] OR Oripavine[tw] OR Oxycodone[tw] OR Oxymorphone[tw] OR Papaveretum[tw] OR Pentazocine[tw] OR pethidin*[tw] OR Phenazocine[tw] OR Phenoperidine[tw] OR phentanyl[tw] OR Phenylpiperidine[tw] OR Piritramide[tw] OR remifentanil[tw] OR Sufentanil[tw] OR sulfentanil[tw] OR sulfentanyl[tw] OR tapentadol[tw] OR Tilidine[tw] OR Tramadol*[tw] OR Actiq[tw] OR adolonta[tw] OR Amadol[tw] OR Anpec[tw] OR Ardinex[tw] OR Asimadolin*[tw] OR Astramorph[tw] OR Avinza[tw] OR Biodalgic[tw] OR Pethidine[tw] OR Carfentanil[tw] OR Codinovo[tw] OR Contramal[tw] OR Demerol[tw] OR Dicodid[tw] OR dihydrohydroxycodeinone[tw] OR dihydromorphinone[tw] OR Dihydrone[tw] OR Dilaudid[tw] OR Dinarkon[tw] OR Dolantin[tw] OR Dolargan[tw] OR Dolcontral[tw] OR Dolosal[tw] OR Dolsin[tw] OR Durogesic[tw] OR Duromorph[tw] or Epimorph[tw] OR Eucodal[tw] OR Exalgo[tw] OR Fentanest[tw] OR Fentora[tw] OR Fortral[tw] OR Hycodan[tw] OR Hycon[tw] OR hydrocodone bitartrate[tw] OR hydroxyacetanilide[tw] OR hydroxycodeinone[tw] OR Isocodeine[tw] OR Isonipecain[tw] OR Jutadol[tw] OR Kadian[tw] OR Dromoran[tw] OR Laudacon[tw] OR Levodroman[tw] OR Levodromoran[tw] OR Levo-dromoran[tw] OR Levorphan[tw] OR Lexir[tw] OR Lidol[tw] OR Lorcet[tw] OR Lortab[tw] OR Lydol[tw] OR Morphia[tw] OR Morphium[tw] OR MS Contin[tw] OR N Methylmorphine[tw] OR Nobligan[tw] OR Norco[tw] OR Numorphan[tw] OR Operidine[tw] OR opiate[tw] OR opioid*[tw] OR Opso[tw] OR Oramorph SR[tw] OR Oripavine[tw] OR Oxecta[tw] OR Oxiconum[tw] OR Oxycodeinon[tw] OR Oxycone[tw] OR Oxycontin[tw] OR Palladone[tw] OR Pancodine[tw] OR Percocet[tw] OR Pethidine[tw] OR Prontofort[tw] OR Propoxyphene[tw] OR Robidone[tw] OR Roxicet[tw] OR Roxicodone[tw] OR Skenan[tw] OR Sublimaze[tw] OR Takadol[tw] OR Talwin[tw] OR Thebaine[tw] OR Theocodin[tw] OR Theradol[tw] OR Tiral[tw] OR Topalgic[tw] OR Tradol[tw] OR Tradolpuren[tw] OR Tradonal[tw] OR Tralgiol[tw] OR Trama[tw] OR Tramadin[tw] OR Tramadoc[tw] OR Trama-Dorsch[tw] OR Tramadura[tw] OR Tramagetic[tw] OR Tramagit[tw] OR Tramake[tw] OR Tramal[tw] OR Tramex[tw] OR Tramundin[tw] OR Trasedal[tw] OR Ultram[tw] OR Vicodin[tw] OR Zamudol[tw] OR Zohydro[tw] OR Zumalgic[tw] OR Zydol[tw] OR Zytram[tw] OR people who inject drug*[tw] OR persons who inject drug*)

AND

Concept #2, SR:

(systematic review [ti] OR meta-analysis [pt] OR meta-analysis [ti] OR systematic literature review [ti] OR this systematic review [tw] OR pooling project [tw] OR (systematic review [tiab] AND review [pt]) OR meta synthesis [ti] OR meta-analy*[ti] OR integrative review [tw] OR integrative research review [tw] OR rapid review [tw] OR umbrella review [tw] OR consensus development conference [pt]

[ta]) OR (clinical guideline [tw] AND management [tw]) OR ((evidence based[ti] OR evidence-based medicine [mh] OR best practice* [ti] OR evidence synthesis [tiab]) AND (review [pt] OR diseases category[mh] OR behavior and behavior mechanisms [mh] OR therapeutics [mh] OR evaluation study[pt] OR validation study[pt] OR guideline [pt] OR pmcbook)) OR ((systematic [tw] OR systematically [tw] OR critical [tiab] OR (study selection [tw]) OR (predetermined [tw] OR inclusion [tw] AND criteri* [tw]) OR exclusion criteri* [tw] OR main outcome measures [tw] OR standard of care [tw] OR standards of care [tw]) AND (survey [tiab] OR surveys [tiab] OR overview* [tw] OR review [tiab] OR reviews [tiab] OR search* [tw] OR handsearch [tw] OR analysis [ti] OR critique [tiab] OR appraisal [tw] OR (reduction [tw] AND (risk [mh] OR risk [tw]) AND (death OR recurrence))) AND (literature [tiab] OR articles [tiab] OR publications [tiab] OR publication [tiab] OR bibliography [tiab] OR bibliographies [tiab] OR published [tiab] OR pooled data [tw] OR unpublished [tw] OR citation [tw] OR citations [tw] OR database [tiab] OR internet [tiab] OR textbooks [tiab] OR references [tw] OR scales [tw] OR papers [tw] OR datasets [tw] OR trials [tiab] OR meta-analy* [tw] OR (clinical [tiab] AND studies [tiab]) OR treatment outcome [mh] OR treatment outcome [tw] OR pmcbook)) NOT(letter [pt] OR newspaper article [pt]) OR "health technology assessment winchester, england"[Journal] OR "Evid Rep Technol Assess (Full Rep)"[Journal] OR "Evid Rep Technol Assess (Summ)"[Journal] OR "Int J Technol Assess Health Care"[Journal] OR "GMS Health Technol Assess"[Journal] OR "Health Technol Assess (Rockv)"[Journal] OR "Health Technol Assess Rep"[Journal]

AND

Concept #3, preventive interventions

("prevention and control" [Subheading] OR "Primary Prevention"[Mesh] OR "Centers for Disease Control and Prevention, U.S."[Mesh] OR "Early Medical Intervention"[Mesh] OR "Internet-Based Intervention "[Mesh] OR "Early Medical Intervention "[Mesh] OR "Early Intervention, Educational "[Mesh] OR "Preventive Health Services"[Mesh] OR "Needle-Exchange Programs"[Mesh] OR "Education"[Mesh] OR "National Health Programs"[Mesh] OR "Diagnostic Screening Programs"[Mesh] OR "Mandatory Programs"[Mesh] OR "Voluntary Programs"[Mesh] OR "Immunization Programs"[Mesh] OR "Regional Medical Programs"[Mesh] OR "Health Promotion"[Mesh] OR "Health Facilities, Proprietary"[Mesh] OR "Referral and Consultation"[Mesh] OR "Prescription Drug Monitoring Programs"[Mesh] OR "Preventive Health Services"[Mesh] OR "Preventive Medicine"[Mesh] OR "Primary Prevention"[Mesh] OR "prevention and control" [Subheading] OR "Public Health"[Mesh] OR "Consumer Health Information"[Mesh] OR "Health Promotion"[Mesh] OR "Community Mental Health Services"[Mesh] OR "Community Health Services"[Mesh] OR "Policy"[Mesh] OR "Public Policy"[Mesh] OR "Health Policy"[Mesh] OR "Drug and Narcotic Control"[Mesh] OR prevention[tw] OR primary prevention[tw] OR control[tw] OR intervention*[tw] OR preventive[tw] OR preventive program*[tw] OR prevention program*[tw] OR government program*[tw] OR policy[tw] OR policies[tw] OR health services[tw] OR screening[tw] OR immunization*[tw] OR health promotion[tw] OR prescription drug monitoring program*[tw] OR maintenance treatment[tw] OR "Opiate Substitution Treatment"[Mesh] OR substitution treatment[tw] OR substitution therapy[tw] OR needle exchange program*[tw] OR needle/syringe program*[tw] OR needle and syringe program*[tw] OR psychosocial intervention*[tw] OR risk-reduction intervention*[tw] OR risk reduction Intervention*[tw] OR behavioural intervention*[tw] OR Public Health Agency of Canada[tw])

AND

Concept #4, infections

(Infections[Mesh] OR Infect*[tw] OR communicable disease*[tw] OR virus disease*[tw] OR viral disease* OR viral illness*[tw] OR viral infection*[tw] OR bacterial infection*[tw])

Filters: Humans

Search strategy, Cochrane

Search Name: narcotic* or opiate* or opioid* or acetylmethadol or alfentanil or anileridine or Benzomorphan* or bezitramide or buprenorphine or butorphanol or Codeine or Dextromethorphan or Dextromoramide or Dextropropoxyphene or dezocine or Diamorphine or dihydrocodein* or Diphenylpropylamine or Ethylmorphine or Fentanyl* or Heroin or Hydrocodon* or Hydromorphon* or ketobemidone or levacetylmethadol or Meperidine or Meptazinol or methadone or Morphan* or Morphin* or nalbuphine or nicomorphine or normethadone or Opium or Oripavine or Oxycodone or Oxymorphone or Papaveretum or Pentazocine or pethidin* or Phenazocine or Phenoperidine or phentanyl or Phenylpiperidine or Piritramide or remifentanil or Sufentanil or sulfentanil or sulfentanyl or tapentadol or Tilidine or Tramadol* or Actiq or adolonta or Amadol or Anpec or Ardinex or Asimadolin* or Astramorph or Avinza or Biodalgic or Pethidine or Carfentanil or Codinovo or Contramal or Demerol or Dicodid or dihydrohydroxycodeinone or dihydromorphinone or Dihydrone or Dilaudid or Dinarkon or Dolantin or Dolargan or Dolcontral or Dolosal or Dolsin or Durogesic or Duromorph or Epimorph or Eucodal or Exalgo or Fentanest or Fentora or Fortral or Hycodan or Hycon or hydrocodone bitartrate or hydroxyacetanilide or hydroxycodeinone or Isocodeine or Isonipecain or Jutadol or Kadian or Dromoran or Laudacon or Levodroman or Levodromoran or Levo-dromoran or Levorphan or Lexir or Lidol or Lorcet or Lortab or Lydol or Morphia or Morphium or MS Contin or N Methylmorphine or Nobligan or Norco or Numorphan or Operidine or opiate or opioid* or Opso or Oramorph SR or Oripavine or Oxecta or Oxiconum or Oxycodeinon or Oxycone or Oxycontin or Palladone or Pancodine or Percocet or Pethidine or Prontofort or Propoxyphene or Robidone or Roxicet or Roxicodone or Skenan or Sublimaze or Takadol or Talwin or Thebaine or Theocodin or Theradol or Tiral or Topalgic or Tradol or Tradolpuren or Tradonal or Tralgiol or Trama or Tramadin or Tramadoc or Trama-Dorsch or Tramadura or Tramagetic or Tramagit or Tramake or Tramal or Tramex or Tramundin or Trasedal or Ultram or Vicodin or Zamudol or Zohydro or Zumalgic or Zydol or Zytram or people who inject drug* or persons who inject drug* in Title Abstract Keyword AND Infect* or communicable disease* or virus disease* or viral disease* or viral illness* or viral infection* or bacterial infection* in Title Abstract Keyword AND prevention or control or intervention* or preventive or (government program*) or policy or policies or (health service*) or screening or immunization* or (health promotion) or (prescription drug monitoring program*) or (maintenance treatment) or (substitution treatment) or (substitution therapy) or (needle exchange program*) or ("needle and syringe program*") or (Public Health Agency of Canada) in Title Abstract Keyword (Word variations have been searched)

ID Search

#1 narcotic* or opiate* or opioid* or acetylmethadol or alfentanil or anileridine or Benzomorphan* or bezitramide or buprenorphine or butorphanol or Codeine or Dextromethorphan or Dextromoramide or Dextropropoxyphene or dezocine or Diamorphine or dihydrocodein* or Diphenylpropylamine or Ethylmorphine or Fentanyl* or Heroin or Hydrocodon* or Hydromorphon* or ketobemidone or levacetylmethadol or Meperidine or Meptazinol or methadone or Morphan* or Morphin* or nalbuphine or nicomorphine or normethadone or Opium or Oripavine or Oxycodone or

Oxymorphone or Papaveretum or Pentazocine or pethidin* or Phenazocine or Phenoperidine or phentanyl or Phenylpiperidine or Piritramide or remifentanil or Sufentanil or sulfentanil or sulfentanyl or tapentadol or Tilidine or Tramadol* or Actiq or adolonta or Amadol or Anpec or Ardinex or Asimadolin* or Astramorph or Avinza or Biodalgic or Pethidine or Carfentanil or Codinovo or Contramal or Demerol or Dicodid or dihydrohydroxycodeinone or dihydromorphinone or Dihydrone or Dilaudid or Dinarkon or Dolantin or Dolargan or Dolcontral or Dolosal or Dolsin or Durogesic or Duromorph or Epimorph or Eucodal or Exalgo or Fentanest or Fentora or Fortral or Hycodan or Hycon or hydrocodone bitartrate or hydroxyacetanilide or hydroxycodeinone or Isocodeine or Isonipecain or Jutadol or Kadian or Dromoran or Laudacon or Levodroman or Levodromoran or Levo-dromoran or Levorphan or Lexir or Lidol or Lorcet or Lortab or Lydol or Morphia or Morphium or MS Contin or N Methylmorphine or Nobligan or Norco or Numorphan or Operidine or opiate or opioid* or Opso or Oramorph SR or Oripavine or Oxecta or Oxiconum or Oxycodeinon or Oxycone or Oxycontin or Palladone or Pancodine or Percocet or Pethidine or Prontofort or Propoxyphene or Robidone or Roxicet or Roxicodone or Skenan or Sublimaze or Takadol or Talwin or Thebaine or Theocodin or Theradol or Tiral or Topalgic or Tradol or Tradolpuren or Tradonal or Tralgiol or Trama or Tramadin or Tramadoc or Trama-Dorsch or Tramadura or Tramagetic or Tramagit or Tramake or Tramal or Tramex or Tramundin or Trasedal or Ultram or Vicodin or Zamudol or Zohydro or Zumalgic or Zydol or Zytram or people who inject drug* or persons who inject drug*:ti,ab,kw AND Infect* or communicable disease* or virus disease* or viral disease* or viral illness* or viral infection* or bacterial infection*:ti,ab,kw AND prevention or control or intervention* or preventive or (government program*) or policy or policies or (health service*) or screening or immunization* or (health promotion) or (prescription drug monitoring program*) or (maintenance treatment) or (substitution treatment) or (substitution therapy) or (needle exchange program*) or ("needle and syringe program*") or (Public Health Agency of Canada):ti,ab,kw (Word variations have been searched)

Database: Embase Classic+Embase, starting in 1947

Search Strategy:

----

1 "systematic review"/ or exp meta analysis/ or (meta analy* or metaanaly* or (systematic adj3 (review or metareview or metasynthesis))).ti. or (cochrane database of systematic reviews or technology assessment*).jw.

2 *infection/ or exp *bacterial infection/ or exp *viral infection/ or (Infect* or communicable disease* or virus disease* or viral disease* or viral illness* or viral infection* or bacterial infection*).ti,ab,kw.

3 exp "prevention and control"/ or public policy/ or (prevention or control or intervention* or preventive or government program* or policy or policies or health services or screening or immuni#ation* or health promotion or prescription drug monitoring program* or ((maintenance or substitution) adj (therap* or treatment*)) or needle exchange program* or "needle and syringe program*" or Public Health Agency of Canada).ti,ab,kw.

4 exp narcotic analgesic agent/ or exp opiate agonist/ or exp narcotic dependence/ or exp injection drug user/ or (narcotic* or opiate* or opioid* or acetylmethadol or alfentanil or anileridine or Benzomorphan* or bezitramide or buprenorphine or butorphanol or Codeine or Dextromethorphan or Dextromoramide or Dextropropoxyphene or dezocine or Diamorphine or dihydrocodein* or Diphenylpropylamine or Ethylmorphine or Fentanyl* or Heroin or Hydrocodon* or Hydromorphon* or ketobemidone or levacetylmethadol or Meperidine or Meptazinol or methadone or Morphan* or Morphin* or nalbuphine or nicomorphine or normethadone or Opium or Oripavine or Oxycodone or Oxymorphone or Papaveretum or Pentazocine or pethidin* or Phenazocine or Phenoperidine or phentanyl or Phenylpiperidine or Piritramide or remifentanil or Sufentanil or sulfentanil or sulfentanyl or tapentadol or Tilidine or Tramadol* or Actiq or adolonta or Amadol or Anpec or Ardinex or Asimadolin* or Astramorph or Avinza or Biodalgic or Pethidine or Carfentanil or Codinovo or Contramal or Demerol or Dicodid or dihydrohydroxycodeinone or dihydromorphinone or Dihydrone or Dilaudid or Dinarkon or Dolantin or Dolargan or Dolcontral or Dolosal or Dolsin or Durogesic or Duromorph or Epimorph or Eucodal or Exalgo or Fentanest or Fentora or Fortral or Hycodan or Hycon or hydrocodone bitartrate or hydroxyacetanilide or hydroxycodeinone or Isocodeine or Isonipecain or Jutadol or Kadian or Dromoran or Laudacon or Levodroman or Levodromoran or Levo-dromoran or Levorphan or Lexir or Lidol or Lorcet or Lortab or Lydol or Morphia or Morphium or MS Contin or N Methylmorphine or Nobligan or Norco or Numorphan or Operidine or opiate or opioid* or Opso or Oramorph SR or Oripavine or Oxecta or Oxiconum or Oxycodeinon or Oxycone or Oxycontin or Palladone or Pancodine or Percocet or Pethidine or Prontofort or Propoxyphene or Robidone or Roxicet or Roxicodone or Skenan or Sublimaze or Takadol or Talwin or Thebaine or Theocodin or Theradol or Tiral or Topalgic or Tradol or Tradolpuren or Tradonal or Tralgiol or Trama or Tramadin or Tramadoc or Trama-Dorsch or Tramadura or Tramagetic or Tramagit or Tramake or Tramal or Tramex or Tramundin or Trasedal or Ultram or Vicodin or Zamudol or Zohydro or Zumalgic or Zydol or Zytram or people who inject drug* or persons who inject drug*).ti,ab,kw.

5 1 and 2 and 3 and 4

6 5 not ((exp animal/ or animal experiment/ or nonhuman/) not (exp human/ or human experiment/))

****

Search strategy, Epistemonikos

1.Opioids

narcotic* or opiate* or opioid* or acetylmethadol or alfentanil or anileridine or Benzomorphan* or bezitramide or buprenorphine or butorphanol or Codeine or Dextromethorphan or Dextromoramide or Dextropropoxyphene or dezocine or Diamorphine or dihydrocodein* or Diphenylpropylamine or Ethylmorphine or Fentanyl* or Heroin or Hydrocodon* or Hydromorphon* or ketobemidone or levacetylmethadol or Meperidine or Meptazinol or methadone or Morphan* or Morphin* or nalbuphine or nicomorphine or normethadone or Opium or Oripavine or Oxycodone or Oxymorphone or Papaveretum or Pentazocine or pethidin* or Phenazocine or Phenoperidine or phentanyl or Phenylpiperidine or Piritramide or remifentanil or Sufentanil or sulfentanil or sulfentanyl or tapentadol or Tilidine or Tramadol* or Actiq or adolonta or Amadol or Anpec or Ardinex or Asimadolin* or Astramorph or Avinza or Biodalgic or Pethidine or Carfentanil or Codinovo or Contramal or Demerol or Dicodid or dihydrohydroxycodeinone or dihydromorphinone or Dihydrone or Dilaudid or Dinarkon or Dolantin or Dolargan or Dolcontral or Dolosal or Dolsin or Durogesic or Duromorph or Epimorph or Eucodal or Exalgo or Fentanest or Fentora or Fortral or Hycodan or Hycon or hydrocodone or hydroxyacetanilide or hydroxycodeinone or Isocodeine or Isonipecain or Jutadol or Kadian or Dromoran or Laudacon or Levodroman or Levodromoran or Levo-dromoran or Levorphan or Lexir or Lidol or Lorcet or Lortab or Lydol or Morphia or Morphium or MS Contin or N Methylmorphine or narcotic or Nobligan or Norco or Numorphan or Operidine or opiate or opioid* or Opso or Oramorph or Oripavine or Oxecta or Oxiconum or Oxycodeinon or Oxycone or Oxycontin or Palladone or Pancodine or Percocet or Pethidine or Prontofort or Propoxyphene or Robidone or Roxicet or Roxicodone or Skenan or Sublimaze or Takadol or Talwin or Thebaine or Theocodin or Theradol or Tiral or Topalgic or Tradol or Tradolpuren or Tradonal or Tralgiol or Trama or Tramadin or Tramadoc or Trama-Dorsch or Tramadura or Tramagetic or Tramagit or Tramake or Tramal or Tramex or Tramundin or Trasedal or Ultram or Vicodin or Zamudol or Zohydro or Zumalgic or Zydol or Zytram or (people who inject drug*) or (injection drug user*)

2.Infections
Infect* or (communicable disease*) or (virus disease*) or (viral disease*) or (viral illness*) or (viral infection*) or (bacterial infection*)

3.Prevention

prevention or control or intervention* or preventive or (government program*) or policy or policies or (health service*) or screening or immunization* or (health promotion) or (prescription drug monitoring program*) or (maintenance treatment) or (substitution treatment) or (substitution therapy) or (needle exchange program*) or ("needle and syringe program*") or (Public Health Agency of Canada)

Filter SR

Filter Interventions was not applied to increased sensitivity

Database: PsycINFO, starting in 1987
Search Strategy:
----
1 (meta analy* or metaanaly* or (systematic adj3 (review or metareview or metasynthesis))).ti. or exp Meta Analysis/ or ("0830" or "1200" or "1300").md.

2 exp Drug Addiction/ or exp Methadone/ or exp Heroin Addiction/ or exp Drug Abuse/ or intravenous drug usage/ or exp narcotic drugs/ or (narcotic* or opiate* or opioid* or acetylmethadol or alfentanil or anileridine or Benzomorphan* or bezitramide or buprenorphine or butorphanol or Codeine or Dextromethorphan or Dextromoramide or Dextropropoxyphene or dezocine or Diamorphine or dihydrocodein* or Diphenylpropylamine or Ethylmorphine or Fentanyl* or Heroin or Hydrocodon* or Hydromorphon* or ketobemidone or levacetylmethadol or Meperidine or Meptazinol or methadone or Morphan* or Morphin* or nalbuphine or nicomorphine or normethadone or Opium or Oripavine or Oxycodone or Oxymorphone or Papaveretum or Pentazocine or pethidin* or Phenazocine or Phenoperidine or phentanyl or Phenylpiperidine or Piritramide or remifentanil or Sufentanil or sulfentanil or sulfentanyl or tapentadol or Tilidine or Tramadol* or Actiq or adolonta or Amadol or Anpec or Ardinex or Asimadolin* or Astramorph or Avinza or Biodalgic or Pethidine or Carfentanil or Codinovo or Contramal or Demerol or Dicodid or dihydrohydroxycodeinone or dihydromorphinone or Dihydrone or Dilaudid or Dinarkon or Dolantin or Dolargan or Dolcontral or Dolosal or Dolsin or Durogesic or Duromorph or Epimorph or Eucodal or Exalgo or Fentanest or Fentora or Fortral or Hycodan or Hycon or hydrocodone bitartrate or hydroxyacetanilide or hydroxycodeinone or Isocodeine or Isonipecain or Jutadol or Kadian or Dromoran or Laudacon or Levodroman or Levodromoran or Levo-dromoran or Levorphan or Lexir or Lidol or Lorcet or Lortab or Lydol or Morphia or Morphium or MS Contin or N Methylmorphine or narcotic or Nobligan or Norco or Numorphan or Operidine or opiate or opioid* or Opso or Oramorph SR or Oripavine or Oxecta or Oxiconum or Oxycodeinon or Oxycone or Oxycontin or Palladone or Pancodine or Percocet or Pethidine or Prontofort or Propoxyphene or Robidone or Roxicet or Roxicodone or Skenan or Sublimaze or Takadol or Talwin or Thebaine or Theocodin or Theradol or Tiral or Topalgic or Tradol or Tradolpuren or Tradonal or Tralgiol or Trama or Tramadin or Tramadoc or Trama-Dorsch or Tramadura or Tramagetic or Tramagit or Tramake or Tramal or Tramex or Tramundin or Trasedal or Ultram or Vicodin or Zamudol or Zohydro or Zumalgic or Zydol or Zytram or people who inject drug* or persons who inject drug*).mp.
3 exp Intervention/ or exp Prevention/ or exp Health Promotion/ or exp Community Services/ or exp Health Care Services/ or exp Immunization/ or exp Harm Reduction/ or exp Needle Exchange Programs/ or exp "Substance Use Treatment"/ or exp Harm Reduction/ or exp Drug Abuse Prevention/ or exp Drug Education/ or exp Screening/ or (prevention or primary prevention or control or intervention* or preventive or preventive program* or prevention program* or government program* or policy or policies or health services or screening or immunization* or health promotion or prescription drug monitoring program* or maintenance treatment or substitution treatment or substitution therapy or needle exchange program* or "needle and syringe program*" or psychosocial intervention* or risk-reduction intervention* or risk reduction Intervention* or behavioural intervention* or Public Health Agency of Canada).mp.
4 exp Infectious Disorders/ or (Infect* or communicable disease* or virus disease* or viral disease* or viral illness* or viral infection* or bacterial infection*).mp. [mp=title, abstract, heading word, table of contents, key concepts, original title, tests & measures, mesh]
5 1 and 2 and 3 and 4

****

Appendix 5.

Methods

SR of SRs methodology. We applied an SR of SRs methodology [1], a subtype of the Overview of Systematic Reviews [2] approach. Both these methods synthesize review-based data, therefore, they are commonly called umbrella reviews. Overview of SRs might not use an exhaustive search strategy and/or a comprehensive set of information sources. Contrary to this, SR of SRs uses a systematic review methodology. This allows performing a comprehensive review of the SR-level of evidence. This methodology is used to inform guidelines and clinical practices [3] as such synthesis eliminates the need for clinicians and decision makers to synthesize the results of multiple SRs on their own [2,4,5].

Our PICO question is "What is the SR-level evidence on the scope and effectiveness of interventions to prevent opioid use associated infections in adults who use legal/illegal opioids as compared to those not participating in an intervention or as compared to the time prior to the intervention implementation?"

Eligibility criteria in a PICO format

Population (P). Included were SRs studying adults who use pharmacological or non-pharmacological opioids, legally or illegally, by any route of use, with no restrictions in terms of settings (e.g., inpatients, outpatients, epidemiological cohorts etc.). SRs with population using unusual or experimental substances, as well as non-opium poppy plants were excluded. SRs evaluating only pediatric populations, including teenagers, were excluded. ***Intervention (I).*** All

interventions (policy, program, behavioural intervention etc.) to prevent acquisition and/or transmission of an opioid use - associated infection were included, counting prevention of co-infections (opioid users with a confirmed infection acquiring another infectious agent). All common infectious diagnoses relevant to opioid use were considered. ***Comparator (C).*** All study types (e.g., experimental or observational) either with a comparator group (opioid users not participating in an intervention / program / not affected by a policy / approach) or time/population prior to implementation of policy (for population-level studies) were included. ***Outcome (O).*** The effectiveness of an intervention (program, policy) to prevent opioid use – associated infections was an outcome. Measures of effectiveness of the intervention (program, policy) were represented by effect measures of an association between the incidence of infection (e.g., HCV/HIV seroconversion) and participation in the intervention. The effect estimates were odds ratio, risk

Grading overall quality of SRs

Overall quality of the results of SRs was graded as either high, moderate quality, low, or critically low. Quality of SR was consequently reflected in the data synthesis. More specifically, reviews were categorized into high quality 'core' reviews, which produced the essential evidence on the effectiveness of interventions, and 'supplementary' reviews, which were considered not being sufficient quality for the authors conclusion to be included in he SR of SRs final conclusions but provided information on primary papers to complement the core reviews. This way of categorization was previously described and used in the published overviews of SRs related to our topic [6,7]. In our SR of SRs, 2 studies [8,9] contained only one original paper relevant to our review question; therefore, we could not define authors' conclusions as an SR-level of synthesized

evidence and categorized this SR as “supplemental” despite these review was of moderate quality. In our SR of SRs, level of evidence was categorized as ‘sufficient’, ‘tentative’, ‘insufficient’ or ‘no’ review-level evidence, using previously developed framework based on the findings of the primary studies included in reviews, and on the conclusions of core review authors [6,10].

1. Bougioukas KI, Liakos A, Tsapas A, Ntzani E, Haidich A-B. Preferred reporting items for overviews of systematic reviews including harms checklist: a pilot tool to be used for balanced reporting of benefits and harms. *Journal of Clinical Epidemiology.* 2018;93:9-24.
2. Pollock M, Fernandes RM, Becker LA, Pieper D, Hartling L. Chapter V: overviews of reviews. *Cochrane Handbook for Systematic Reviews of Interventions version.* 2018;6.
3. Worswick J, Wayne SC, Bennett R, et al. Improving quality of care for persons with diabetes: an overview of systematic reviews-what does the evidence tell us? *Systematic reviews.* 2013;2(1):26.
4. Aromataris E, Fernandez R, Godfrey CM, Holly C, Khalil H, Tungpunkom P. Summarizing systematic reviews: methodological development, conduct and reporting of an umbrella review approach. *Int J Evid Based Healthc.* 2015;13(3):132-140.
5. Hartling L, Chisholm A, Thomson D, Dryden DM. A descriptive analysis of overviews of reviews published between 2000 and 2011. *PloS one.* 2012;7(11):e49667.
6. Palmateer N, Kimber J, Hickman M, Hutchinson S, Rhodes T, Goldberg D. Evidence for the effectiveness of sterile injecting equipment provision in preventing hepatitis C and human immunodeficiency virus transmission among injecting drug users: A review of reviews. *Addiction.* 2010;105(5):844-859.
7. MacArthur GJ, van Velzen E, Palmateer N, et al. Interventions to prevent HIV and hepatitis C in people who inject drugs: a review of reviews to assess evidence of effectiveness. *International Journal of Drug Policy.* 2014;25(1):34-52.
8. Underhill K, Dumont D, Operario D. HIV prevention for adults with criminal justice involvement: A systematic review of HIV risk-reduction interventions in incarceration and community settings. *American Journal of Public Health.* 2014;104(11):e27-e53.
9. Jones L, Pickering L, Sumnall H, McVeigh J, Bellis MA. A review of the effectiveness and cost-effectiveness of needle and syringe programmes for injecting drug users. *Centre for Pulic Health, Liverpool John Moores University.* 2008.
10. Ellis S, Barnett-Page E, Morgan A, Taylor L, Walters R, Goodrich J. HIV prevention: a review of reviews assessing the effectiveness of interventions to reduce the risk of sexual transmission. Health Development Agency London; 2003.
