# Supplementary figures and images for: Effectiveness of Interventions for Prevention of Common Infections Among Opioid Users: A Systematic Review of Systematic Reviews
Source: Front Public Health. 2022 Feb 22;10:749033. doi: 10.3389/fpubh.2022.749033 (PMC8901608; doi:10.3389/fpubh.2022.749033)

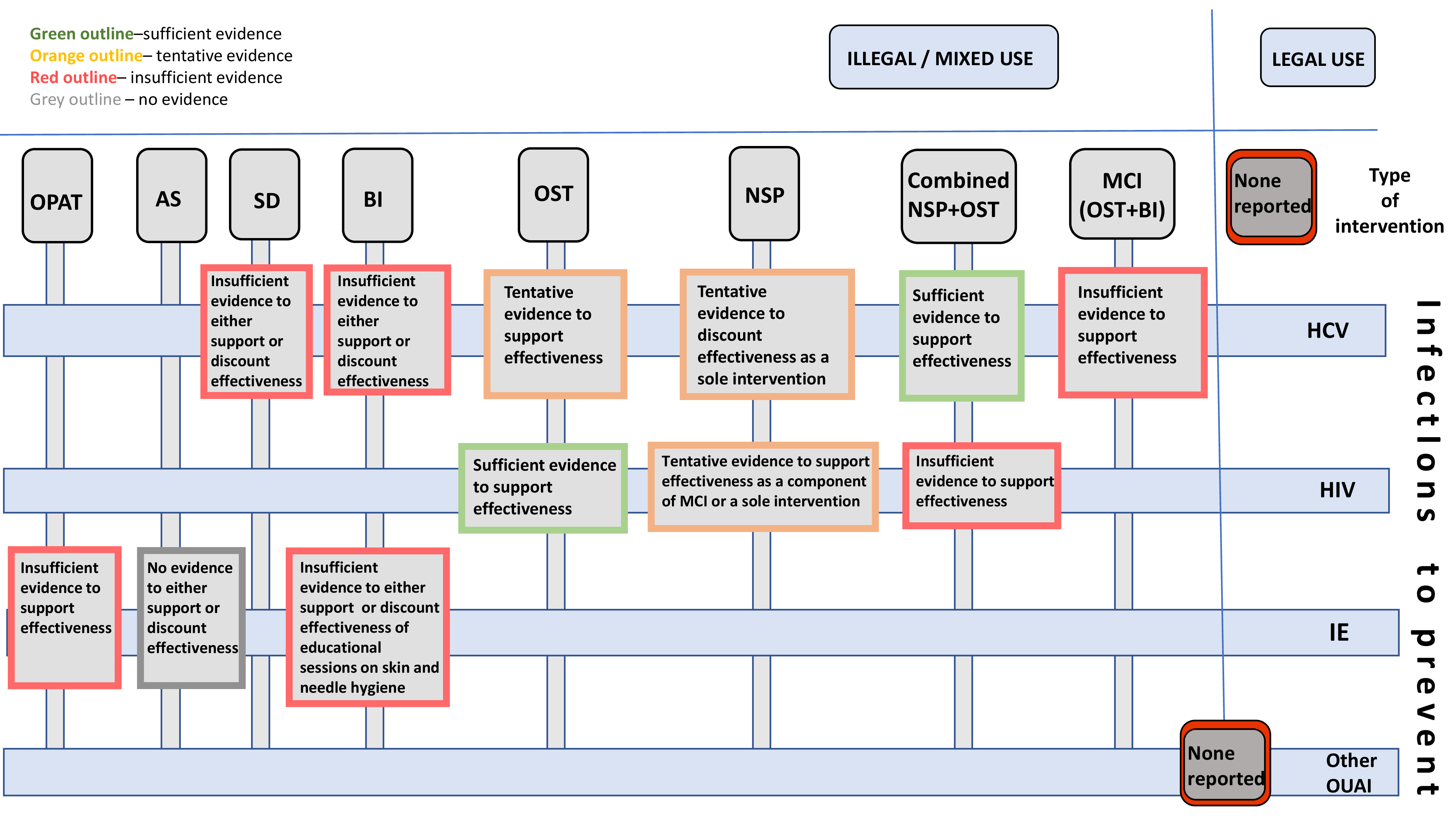

Supplement: Supplementary Figure 1 — Graphical abstract: Effectiveness of interventions to prevent infections in persons who use opioids: existing evidence and knowledge gaps. [file Image_1.TIF]
